# Tobacco Root Endophytic *Arthrobacter* Harbors Genomic Features Enabling the Catabolism of Host-Specific Plant Specialized Metabolites

**DOI:** 10.1128/mBio.00846-21

**Published:** 2021-05-28

**Authors:** Tomohisa Shimasaki, Sachiko Masuda, Ruben Garrido-Oter, Takashi Kawasaki, Yuichi Aoki, Arisa Shibata, Wataru Suda, Ken Shirasu, Kazufumi Yazaki, Ryohei Thomas Nakano, Akifumi Sugiyama

**Affiliations:** a Research Institute for Sustainable Humanosphere, Kyoto University, Uji, Japan; b Plant Immunity Research Group, RIKEN Center for Sustainable Resource Science, Yokohama, Kanagawa, Japan; c Department of Plant Microbe Interactions, Max Planck Institute for Plant Breeding Research, Cologne, Germany; d Cluster of Excellence on Plant Sciences (CEPLAS), Max Planck Institute for Plant Breeding Research, Cologne, Germany; e Tohoku Medical Megabank Organization, Tohoku University, Sendai, Japan; f Laboratory for Microbiome Sciences, RIKEN Center for Integrative Medical Sciences, Yokohama, Japan; University of Toronto

**Keywords:** root bacterial microbiota, plant specialized metabolite, Amadori-type opine, comparative genomics analysis, alkaloids

## Abstract

Plant roots constitute the primary interface between plants and soilborne microorganisms and harbor microbial communities called the root microbiota. Recent studies have demonstrated a significant contribution of plant specialized metabolites (PSMs) to the assembly of root microbiota. However, the mechanistic and evolutionary details underlying the PSM-mediated microbiota assembly and its contribution to host specificity remain elusive. Here, we show that the bacterial genus *Arthrobacter* is predominant specifically in the tobacco endosphere and that its enrichment in the tobacco endosphere is partially mediated by a combination of two unrelated classes of tobacco-specific PSMs, santhopine and nicotine. We isolated and sequenced *Arthrobacter* strains from tobacco roots as well as soils treated with these PSMs and identified genomic features, including but not limited to genes for santhopine and nicotine catabolism, that are associated with the ability to colonize tobacco roots. Phylogenomic and comparative analyses suggest that these genes were gained in multiple independent acquisition events, each of which was possibly triggered by adaptation to particular soil environments. Taken together, our findings illustrate a cooperative role of a combination of PSMs in mediating plant species-specific root bacterial microbiota assembly and suggest that the observed interaction between tobacco and *Arthrobacter* may be a consequence of an ecological fitting process.

## INTRODUCTION

Plant roots secrete their photosynthates to the rhizosphere, a fraction of soil surrounding the root (1), creating a nutrient-rich environment with a distinctive metabolic profile compared to the bulk soil (2, 3). These rhizosphere metabolites attract or repel particular soil bacteria, resulting in characteristic bacterial communities in the rhizosphere. A subset of rhizosphere bacteria can further colonize the root surface and the interior, constituting the rhizoplane and endosphere bacterial communities, respectively. Bacterial communities on the root surface and in the root interior are collectively called the root bacterial microbiota (4). Four bacterial phyla, *Proteobacteria*, *Actinobacteria*, *Bacteroidetes*, and *Firmicutes*, dominate the root microbiota (5–7), whereas each plant species harbors a different composition of root microbiota at lower taxonomic levels (8), suggesting the ability of plants to modulate root bacterial microbiota in a manner specific to each plant lineage.

Plant specialized metabolites (PSMs), also known as plant secondary metabolites, play an important role in the interaction between the host and its root microbiota, affecting its taxonomic composition (9, 10). For instance, the disruption of the triterpene or coumarin biosynthetic pathway in Arabidopsis thaliana resulted in different taxonomic structures of the root microbiota (11–14). Soil treatment with isoflavone or soyasaponin, two major PSMs in soybean root exudates, altered the compositions of the soil bacterial community and enriched bacteria commonly found in soybean roots (15, 16). Recent large-scale comparative genomics analyses also showed that the bacterial species inhabiting plant tissues had acquired specific metabolic capacities, which enabled their adaptation to plants (17). However, the mechanisms by which PSMs affect the relative abundance of a given bacterial species and whether single or multiple classes of PSMs are needed to shape the microbial community remain unclear. The evolutionary processes through which plants and bacteria establish such PSM-mediated interactions also remain to be addressed.

**FIG 1:**
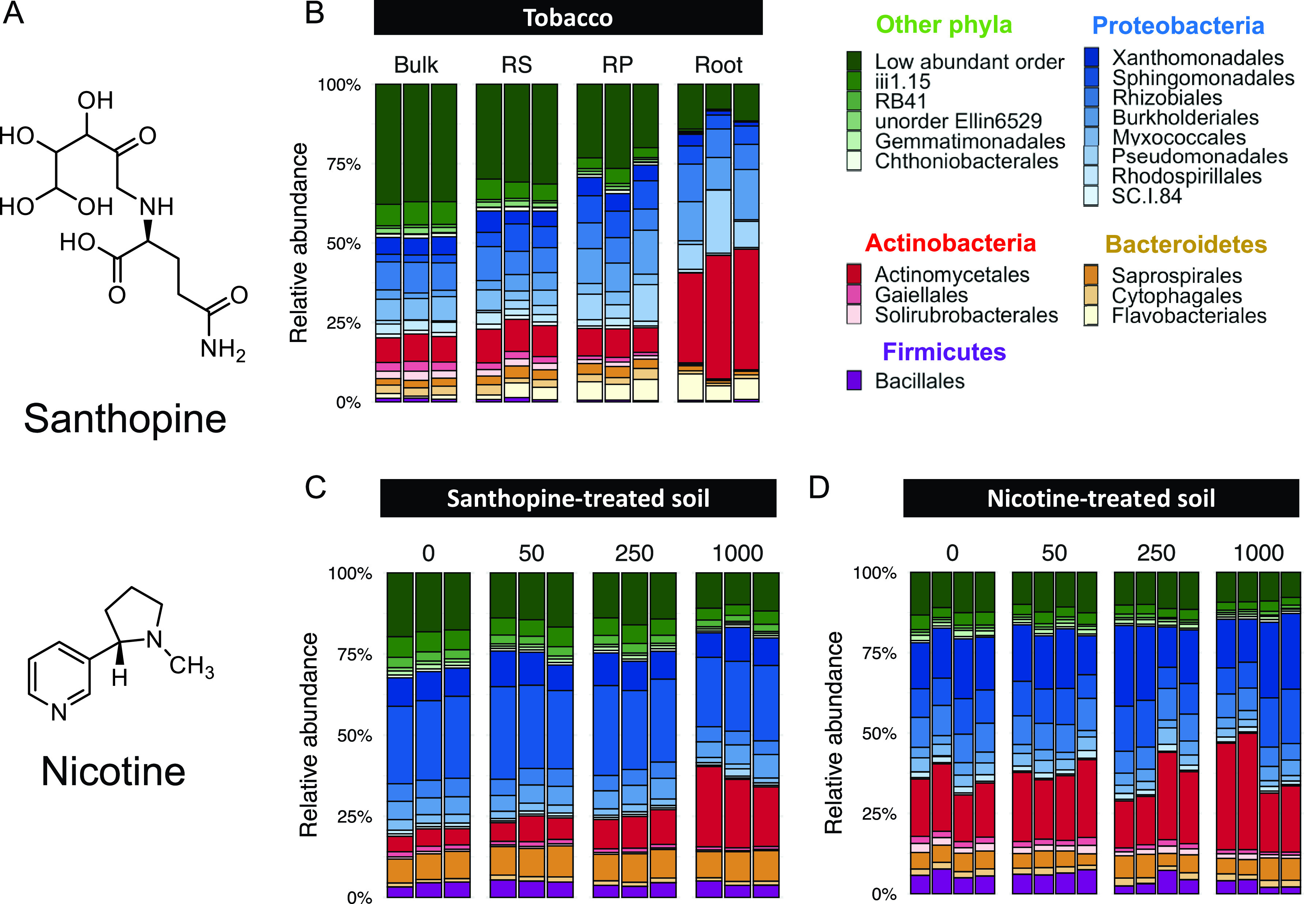
Bacterial community composition in tobacco roots and santhopine/nicotine-treated soil. (A) Chemical structure of santhopine and nicotine. (B to D) Compositions of the 20 most abundant bacterial orders across samples in tobacco roots (B), santhopine-treated soil (C), and nicotine-treated soil (D). Bacterial communities in soils were treated with 50, 250, and 1,000 nmol g^−1^ soil of santhopine or nicotine. Bulk, bulk soil; RS, rhizosphere; RP, rhizoplane; ES, endosphere.

Nicotiana tabacum (cultivated tobacco) is an industrially important crop species and a useful system to study PSM-mediated plant-microbiota interactions, as its PSM profiles, including alkaloids, terpenoids, flavonoids, and aromatic compounds, have been well characterized partly because of their impacts on the flavor of cigarettes (18, 19). The PSM profile of tobacco roots is characterized by a set of tobacco-specific PSMs, such as santhopine and nicotine (Fig. 1A), both of which have potential relevance to the interaction with surrounding microbes (20, 21). Santhopine is an opine whose biosynthesis is induced in crown gall tumors and hairy roots upon infection by pathogenic *Rhizobium* strains (formerly known as the independent genus *Agrobacterium*) (22, 23). During the diversification of the *Nicotiana* genus, some of its species acquired genes for opine biosynthesis, including the mannopine synthase 2 (*MAS2*) gene encoding the enzyme for santhopine synthesis, likely via a horizontal gene transfer (HGT) event from *Rhizobium* species (24–26). N. tabacum then inherited the *MAS2* gene from its parental wild species Nicotiana tomentosiformis to synthesize santhopine at various concentrations in roots, mostly below 1 μmol gFW (fresh weight)^−1^ (20, 27). Opine-catabolic genes have been found in a limited set of bacterial orders, such as *Rhizobiales*, *Actinomycetales*, and *Enterobacteriales* (28), suggesting that opines may serve as nutrients for specific groups of bacteria. In contrast, nicotine, a major alkaloid commonly produced by the *Nicotiana* species at approximately 0.5 μmol gFW^−1^ (29), exhibits strong toxicity and contributes to chemical defense against insect predators (30–32). Nicotine is also catabolized by several bacterial species (33, 34), implying the possible involvement of both santhopine and nicotine in the interaction between tobacco and its root-associated microbiota.

In this study, we analyzed the bacterial communities of the tobacco endosphere and the effect of santhopine and nicotine on the soil bacterial community. This led us to focus on the genus *Arthrobacter*, which was specifically enriched in the tobacco endosphere and PSM-treated soils. By integrating culture-dependent characterization and comparative genomics of *Arthrobacter* isolates, here, we propose a model that explains a process of host-specific root microbiota assembly partially mediated by bacterial catabolism of a combination of PSMs synthesized by the host plant.

## RESULTS

### *Arthrobacter* is predominantly found in the tobacco endosphere and enriched in soils by santhopine and nicotine treatments.

**FIG 2:**
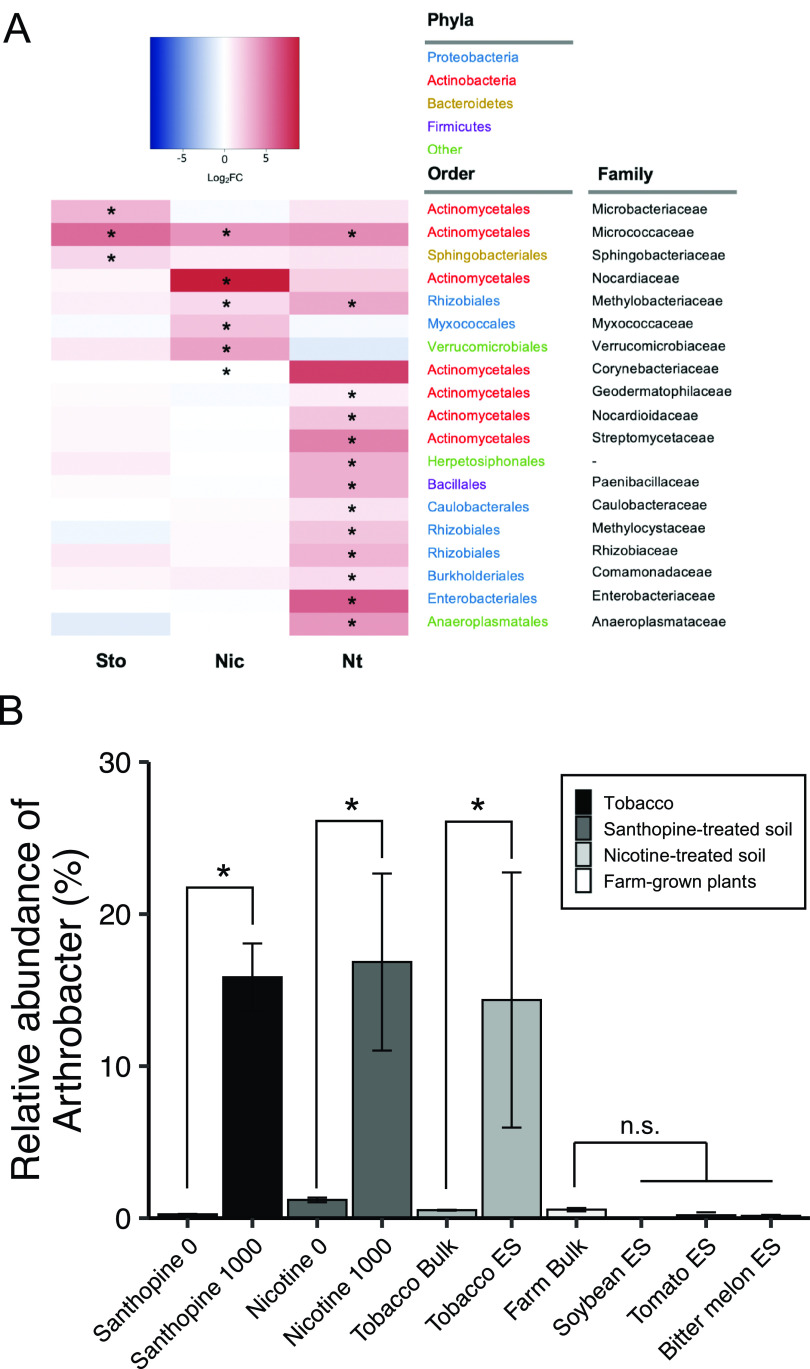
Differential abundance analysis of each sample. (A) Heat map showing the fold changes at the log_2_ scale in the tobacco endosphere and santhopine- and nicotine-treated soils at the highest concentrations, compared to rhizoplane, santhopine mock, and nicotine mock treatments, respectively. Asterisks indicate statistical significance corresponding to the GLM analysis within each data set (α = 0.05). Nic, nicotine-treated soil; Sto, santhopine-treated soil; Nt, tobacco endosphere; FC, fold change. (B) Mean relative abundance of the genus *Arthrobacter* in soils treated with santhopine or nicotine and in the tobacco, tomato, bitter melon, and soybean root endospheres along with their respective bulk soil samples. Error bars represent standard deviations (*n* = 3 for tobacco roots and santhopine-treated soil, and *n* = 4 for nicotine-treated soil). Asterisks indicate statistical significance corresponding to the GLM analysis within each data set (α = 0.05). n.s., not significant.

To investigate the taxonomic composition of the bacterial community associated with tobacco roots by bacterial 16S rRNA gene amplicon sequencing, we employed N. tabacum cv. Burley 21, which is among the cultivars that most strongly express *MAS2* (20). Analysis of taxonomic profiles across samples by fitting normalized read counts to a generalized linear model (GLM) with a negative binomial distribution revealed that the bacterial members belonging to the order *Actinomycetales* were most significantly enriched in the endosphere compared to the rhizosphere and rhizoplane fractions (false discovery rate-corrected *P* values of 6.39 × 10^−12^ and 1.59 × 10^−10^, respectively) (Fig. 1B). In parallel, to identify bacterial taxa that are directly targeted by santhopine and nicotine, we treated soils with either of these metabolites at three different concentrations and compared the compositions of the bacterial community. Permutational analysis of variance (PERMANOVA) revealed that santhopine and nicotine treatments shifted the bacterial community compositions (*P* = 1.00 × 10^−2^ and 5.30 × 10^−2^, respectively) (Fig. 1C and D), resulting in community compositions that were more similar to those of the tobacco endosphere than to those of the bulk soil (see Fig. S1A and B in the supplemental material). We found that the *Micrococcaceae* family was the only family enriched both in the tobacco endosphere compared to the rhizoplane and commonly by santhopine and nicotine treatments (Fig. 2A and Data Set S1) in a dose-dependent manner (Fig. S1C and D). All sequence reads classified as belonging to the family *Micrococcaceae* were assigned to the genus *Arthrobacter*, which represented up to 15% of the total bacterial sequences in the tobacco endosphere and soils treated with santhopine or nicotine (Fig. 2B). Notably, enrichment of *Arthrobacter* in the endosphere is unique to tobacco plants and was not observed in tomato (Solanum lycopersicum), soybean (Glycine max), or bitter melon (Momordica charantia) plants grown in the field where we collected our soil samples (Fig. 2B). Given that the production of santhopine and nicotine is specific to tobacco plants, these results suggest a crucial role of these metabolites in forming tobacco-specific root microbiota structures.

10.1128/mBio.00846-21.2 FIG S1 Bacterial community analysis of santhopine- and nicotine-treated soils. Bacterial communities in santhopine- and nicotine-treated soils were compared with those of the tobacco endosphere. (A) Weighted UniFrac distances between santhopine mock treatment and the bulk soil/endosphere of tobacco and between santhopine treatment at 1,000 nmol g^−1^ soil and the bulk soil/endosphere of tobacco. (B) Weighted UniFrac distances between nicotine mock treatment and the bulk soil/endosphere of tobacco and between nicotine treatment at 1,000 nmol g^−1^ soil and the bulk soil/endosphere of tobacco. Different letters indicate statistical differences corresponding to a Tukey honestly significant difference (HSD) test (*P < *0.05). Bulk, bulk soil; ES, endosphere. (C and D) Mean relative abundances of the genus *Arthrobacter* in santhopine (C)- and nicotine (D)-treated soils. Error bars represent standard deviations (*n* = 3 for santhopine-treated soil, and *n* = 4 for nicotine-treated soil). Download FIG S1, PDF file, 0.08 MB. Copyright © 2021 Shimasaki et al. 2021 Shimasaki et al. https://creativecommons.org/licenses/by/4.0/ This content is distributed under the terms of the Creative Commons Attribution 4.0 International license.

10.1128/mBio.00846-21.8 DATA SET S1 List of bacterial genera significantly enriched in the tobacco endosphere (A), santhopine-treated soil (B), nicotine-treated soil (C), and dual-metabolite-treated soil (D). Download Data Set S1, XLSX file, 0.01 MB. Copyright © 2021 Shimasaki et al. 2021 Shimasaki et al. https://creativecommons.org/licenses/by/4.0/ This content is distributed under the terms of the Creative Commons Attribution 4.0 International license.

### Predominance of a monophyletic clade of the genus *Arthrobacter* in the tobacco endosphere.

We then isolated 252 individual bacterial strains from surface-sterilized tobacco roots, which corresponded to the endosphere fraction in the community profiling experiments, as well as from santhopine- or nicotine-treated soil, including 131 *Arthrobacter* strains, based on their partial 16S rRNA gene sequences (Data Set S2A-C). Fifty-four isolates were then randomly selected for phylogenetic characterization using their nearly complete 16S rRNA gene sequences, which included strains from two other bacterial genera, *Paenarthrobacter* and *Pseudarthrobacter*, recently reclassified from the genus *Arthrobacter* based on their chemotaxonomic traits (35, 36) (Fig. S2). Given that these strains were found to be closely related to other *Arthrobacter* isolates at the 16S rRNA gene sequence level as well as the whole-genome level (see below), these genera are referred to here as part of the single genus *Arthrobacter*. Although the isolates from santhopine- and nicotine-treated soils were relatively diverse in their 16S rRNA gene sequences (Fig. S2), most isolates from tobacco roots were closely related to each other (27 of 30), forming a monophyletic clade with Arthrobacter nicotinovorans DSM420, together with two isolates derived from santhopine-treated soil (Fig. S2). These results indicate the predominance of the tobacco endosphere by a taxonomically limited range of isolates within the genus *Arthrobacter*, and this cannot be explained by the presence of santhopine or nicotine in root tissues alone given the difference between strains isolated from tobacco roots and those isolated from soils treated with these metabolites.

10.1128/mBio.00846-21.3 FIG S2 MLE phylogeny of *Arthrobacter* isolates based on their nearly complete 16S rRNA gene sequences. The phylogenetic tree was constructed by an MLE method. Bootstrap values (1,000 replicates) above 0.6 are shown in nodes. The red branch represents a monophyletic clade containing almost all isolates from tobacco roots as well as the A. nicotinovorans strain DSM420. The name of each strain indicates the origin of bacterial isolates (NtRoot, tobacco roots; StoSoil, santhopine-treated soil; NicSoil, nicotine-treated soil). The isolates subjected to whole-genome sequencing are marked with arrowheads. Download FIG S2, PDF file, 0.08 MB. Copyright © 2021 Shimasaki et al. 2021 Shimasaki et al. https://creativecommons.org/licenses/by/4.0/ This content is distributed under the terms of the Creative Commons Attribution 4.0 International license.

10.1128/mBio.00846-21.9 DATA SET S2 (A to E) Summary data for bacterial isolates from tobacco roots (A), santhopine-treated soil (B), nicotine-treated soil (C), dual-metabolite-treated soil (D), and mock-treated soil (E). (F and G) General genomic features of newly sequenced *Arthrobacter* isolates (F) and classification of the isolation sources of *Arthrobacter* genomes for comparative genomics (G). Assembly qualities were calculated using the DDBJ Fast Annotation and Submission Tool (DFAST) (https://dfast.nig.ac.jp). Download Data Set S2, XLSX file, 0.02 MB. Copyright © 2021 Shimasaki et al. 2021 Shimasaki et al. https://creativecommons.org/licenses/by/4.0/ This content is distributed under the terms of the Creative Commons Attribution 4.0 International license.

### Subspeciation of the genus *Arthrobacter* driven by a whole-genome-scale rearrangement.

**FIG 3:**
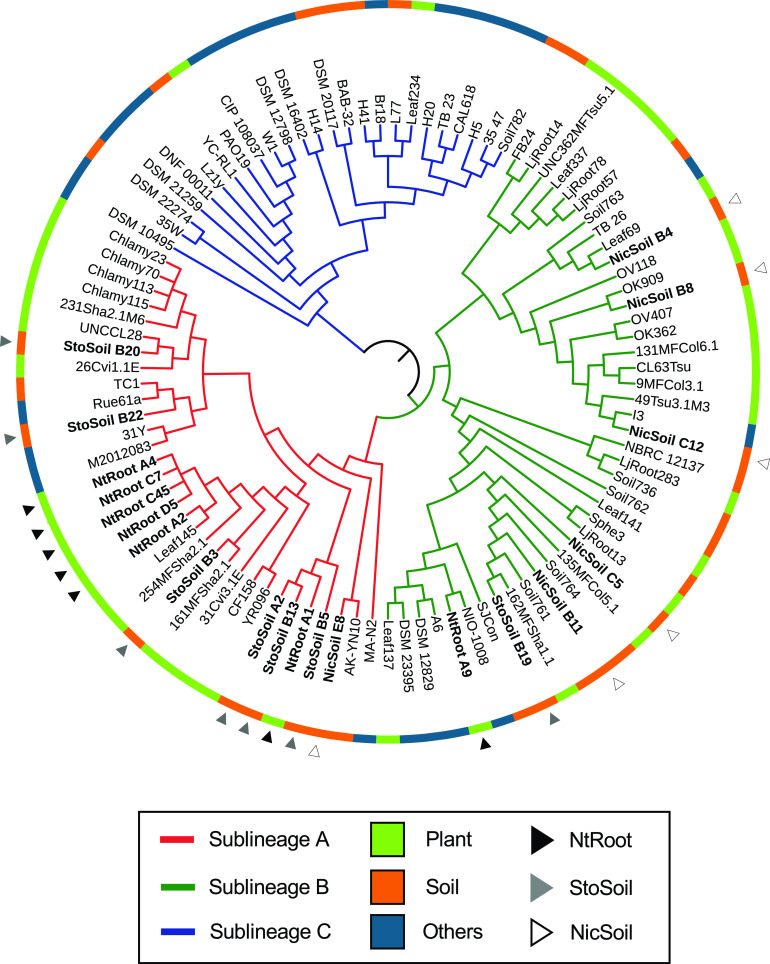
Whole-genome-based phylogenomic reconstruction of the genus *Arthrobacter*. The phylogenetic tree of 99 *Arthrobacter* genomes was inferred from aligned single-copy, vertically inherited marker genes using an MLE method. The branch colors represent the sublineages. The outer ring depicts the isolation source of each genome. Arrowheads indicate the newly sequenced isolates, whose colors represent the origin of each strain.

We hypothesized that the specific enrichment of this particular subset of *Arthrobacter* isolates in the tobacco endosphere is mediated by whole-genome-scale functional properties specific to these isolates. To test this, a taxonomically diverse set of *Arthrobacter* strains (20 strains in total) (Fig. S2) was selected from our culture collection for whole-genome sequencing. We obtained high-quality, closed genomic sequences (Data Set S2F), which were then compared to the genome sequences of 79 previously sequenced *Arthrobacter* isolates obtained from a variety of other plant hosts, soils, and environments (17, 37–39) (Data Set S2G). First, their phylogenetic relationship was inferred based on a set of vertically inherited, single-copy genes based on automated pipeline for phylogenomic analysis (AMPHORA) (40). This analysis defined three distinct phylogroups within the genus *Arthrobacter*, referred to here as sublineages A to C (Fig. 3). All isolates from tobacco roots (NtRoot) and santhopine-treated soil (StoSoil) except for NtRootA9 and StoSoilB19 belonged to sublineage A, and the rest, including five strains from nicotine-treated soil (Nicsoil) were classified into sublineage B. Classification of 131 strains that we isolated from roots or soils (Data Set S2A-C) into these sublineages based on their partial 16S rRNA gene sequences also revealed that tobacco root-derived isolates mainly belonged to sublineage A (*P* = 1.90 × 10^−8^ by Fisher’s exact test). None of the newly sequenced isolates belonged to sublineage C. We classified each genome as plant associated (“plant”), soil derived (“soil”), or derived from other environment sources (“other”), based on their origin of isolation (17). Sublineages A and B were overrepresented by the plant- and soil-associated isolates (*P *= 0.047 and *P* = 2.69 × 10^−3^, respectively, by hypergeometric tests). In contrast, sublineage C was mainly composed of isolates from other environments (*P* = 5.41 × 10^−7^ by a hypergeometric test), such as animal, food, water, and extreme environments (Fig. 3, outer ring).

**FIG 4:**
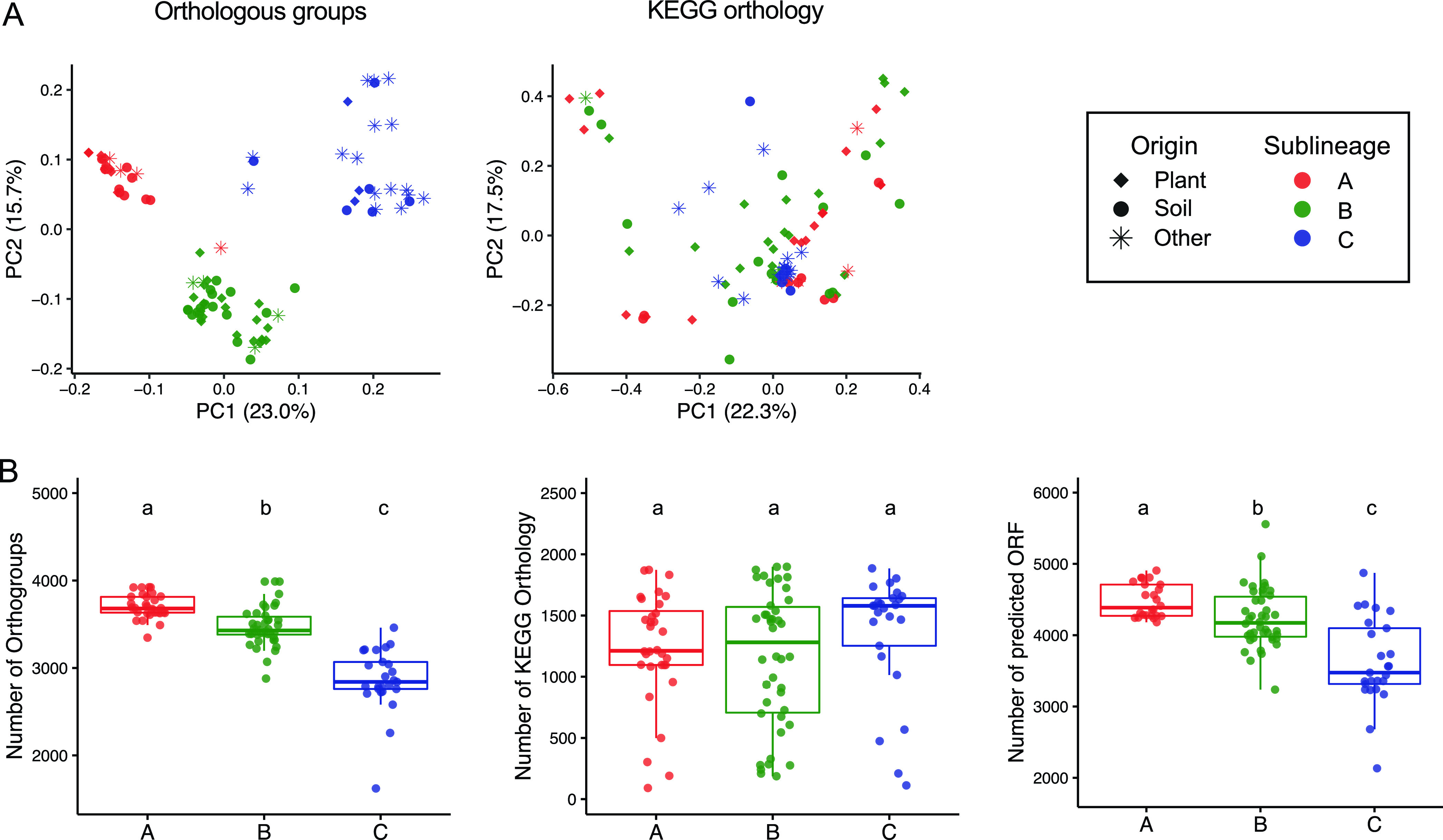
Functional diversity analysis and genomic size comparison between *Arthrobacter* genomes. (A) PCoA plots projecting functional distances between 99 *Arthrobacter* genomes based on the predicted OGs and the KOs. Each point represents each genome. Shapes correspond to the isolation sources, and colors represent the sublineages defined by their AMPHORA phylogeny. (B) Genome size comparison of each *Arthrobacter* sublineage based on the numbers of predicted ORFs, OGs, and KOs. Different letters indicate statistical differences corresponding to a pairwise Wilcoxon rank sum test (*P < *0.05).

To assess the functional potential of these sequenced strains, 99 representative genomes were annotated using the Kyoto Encyclopedia of Genes and Genomes (KEGG) database, resulting in 3,803 KEGG Orthology groups (KOs). We also employed a *de novo* orthology prediction algorithm (41) and obtained 12,020 orthologous groups (OGs), the majority of which had no assigned functional prediction. Principal coordinates analysis (PCoA) was then performed using whole-genome-level functional distances based on the presence or absence of OGs or KOs (38). This showed that the functional distances based on *de novo* OGs, including genes without functional annotations, were tightly associated with the speciation of the sublineages defined by their AMPHORA phylogeny (*R*^2^ = 0.350 and *P < *0.001 by PERMANOVA) (Fig. 4A), while the functional distances based on the annotated KOs resulted in a single large cluster of genomes and did not strongly correlate with the subspeciation events (*R*^2^ = 0.087 and *P < *0.001 by PERMANOVA) (Fig. 4A), suggesting that the diversification of these lineages is found primarily in the less-efficiently annotated portion of their pangenome. Strain MA-N2 belonged to sublineage A based on the phylogenetic analysis (Fig. 3), whereas it was clustered with sublineage B isolates in the PCoA plot of the genomic composition (Fig. 4A), which may be explained by an intermediate status of this strain between sublineages A and B. Based on its encoded functional capabilities, we decided to classify this strain as a constituent of sublineage B for the subsequent genomic analyses. Notably, the numbers of open reading frames (ORFs) and OGs predicted in the genomes from sublineage A were significantly higher than those from sublineages B and C, with sublineage C having the lowest numbers, while the numbers of KOs were comparable among sublineages (Fig. 4B). The larger genome size of plant-associated than non-plant-associated bacteria was also previously observed in a taxonomically broader set of bacterial genomes (17). Overall, these results suggest that the subspeciation of the *Arthrobacter* genus is linked to adaptation to different environments and might have been associated with gains of genes in sublineages A and B and/or losses in sublineage C, whose functional characteristics are yet underexplored.

### *Arthrobacter* sublineage A has unique genetic components.

**FIG 5:**
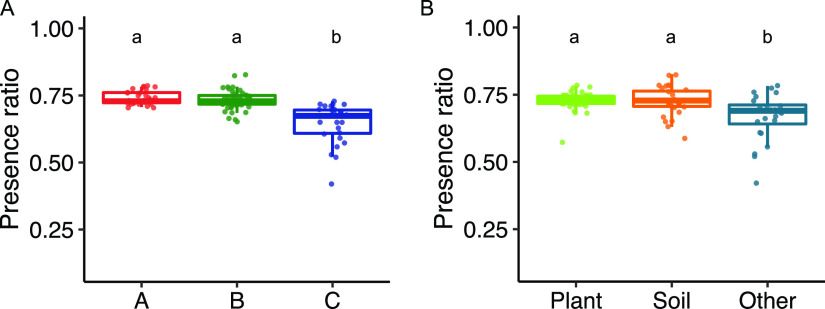
Proportion of rhizobial genes relevant for root colonization. For the genes needed for root colonization, the proportion of the number of genes present in each genome to the total number of genes present in at least one of these genomes (92 genes) was computed and is shown as a boxplot, comparing between sublineages (A) and isolation origins (B). Different letters indicate statistical differences corresponding to a pairwise Wilcoxon rank sum test (*P < *0.05).

To assess whether *Arthrobacter* sublineage A has genomic signatures linked with a plant-associated or endophytic lifestyle, we surveyed the genomes for the prevalence of genes previously identified as being relevant for root colonization in well-characterized rhizobial endophytes (42, 43). Among 157 genes reported in rhizobia, 92 genes were identified in at least one of the genomes in the genus *Arthrobacter* (Fig. S3 and Data Set S3A and B). The proportion of the genes relevant for root colonization found in the genomes from sublineages A and B was significantly higher than that in the genomes from sublineage C, although it was not significantly different between sublineages A and B (Fig. 5A). A similar proportion of genes required for root colonization was also detected between plant- and soil-derived isolates, while plant-derived strains had significantly higher proportions than environment-derived strains (Fig. 5B). This revealed that sublineages A and B share certain genomic features associated with root colonization, which supports the idea that both of these sublineages are better adapted to the plant niche but does not explain the difference between these sublineages in interaction with tobacco roots.

10.1128/mBio.00846-21.4 FIG S3 Phylogenetic distribution of *soc*, *nic*, and genes relevant for root colonization. The tree indicates the AMPHORA phylogeny. The branch colors represent the sublineages. The newly sequenced *Arthrobacter* isolates are represented by boldface type and the first strip. The second strips indicate the isolation origins of each strain. Letters at nodes indicate the most recent common ancestor of each sublineage that was used for the ancestral character estimation. The presence and absence of *soc* and *nic* genes and the genes required for root colonization in symbiotic nodulating rhizobia (Ensifer meliloti 1021 and Rhizobium leguminosarum bv. *viciae* Rlv3841) are represented by closed and open circles, respectively. Download FIG S3, PDF file, 0.7 MB. Copyright © 2021 Shimasaki et al. 2021 Shimasaki et al. https://creativecommons.org/licenses/by/4.0/ This content is distributed under the terms of the Creative Commons Attribution 4.0 International license.

10.1128/mBio.00846-21.10 DATA SET S3 List of rhizobial genes identified in the genus *Arthrobacter* and ancestral components of each *Arthrobacter* sublineage. (A and B) Rhizobial genes were retrieved from E. meliloti 1021 (42) (A) and R. leguminosarum bv. *viciae* Rlv3841 (43) (B). (C and D) Ancestral components were estimated based on the KO profiles unique to sublineage A (C) and sublineage A/B (D). (E to H) Ancestral components were estimated based on the OG profiles unique to sublineage A (E), sublineage B (F), sublineage C (G), and sublineage A/B (H). Download Data Set S3, XLSX file, 0.06 MB. Copyright © 2021 Shimasaki et al. 2021 Shimasaki et al. https://creativecommons.org/licenses/by/4.0/ This content is distributed under the terms of the Creative Commons Attribution 4.0 International license.

To further disentangle the functional differences between sublineages A and B at the genome level, the ancestral characters of each *Arthrobacter* sublineage were estimated based on KO or OG profiles by a maximum likelihood estimation (MLE) approach. Using this analysis, we found 35 (KO) and 380 (OG) ancestral characters unique to sublineages A and B (Fig. S4 and Data Set SC-G). Among those genes, we identified genes known to be involved in root endophytic colonization, such as chemotaxis/bacterial motility, quorum sensing, and secretion systems (44), further supporting the notion that sublineages A and B are better adapted to the plant environment. We also identified 27 (KO) and 349 (OG) ancestral characters unique to sublineage A (Fig. S4 and Data Set S3C). Most ancestral KOs specific to sublineage A (27 KOs with 50 KEGG functional assignments) were functionally categorized as part of carbohydrate and amino acid metabolism (6 and 8 genes, respectively) and the biosynthesis of bacterial secondary metabolites (5 genes). These findings point to the genomic traits unique to sublineage A that contribute to its distinctive biological properties compared to sublineage B.

10.1128/mBio.00846-21.5 FIG S4 Comparison of ancestral characters of each *Arthrobacter* sublineage. Venn diagrams compare the identified ancestral components based on KO (A) and OG (B) profiles. Download FIG S4, PDF file, 0.3 MB. Copyright © 2021 Shimasaki et al. 2021 Shimasaki et al. https://creativecommons.org/licenses/by/4.0/ This content is distributed under the terms of the Creative Commons Attribution 4.0 International license.

### Endophytically colonizing *Arthrobacter* strains possess unique catabolic capacities.

Most bacterial strains isolated from tobacco roots formed a monophyletic clade within sublineage A (Fig. S2), indicating that additional genetic features acquired after the divergence from sublineage B led to the tobacco-specific endophytic competence of these *Arthrobacter* isolates. To assess this, we surveyed the presence of the catabolic genes for santhopine and nicotine, both of which are characteristic of tobacco roots.

**FIG 6:**
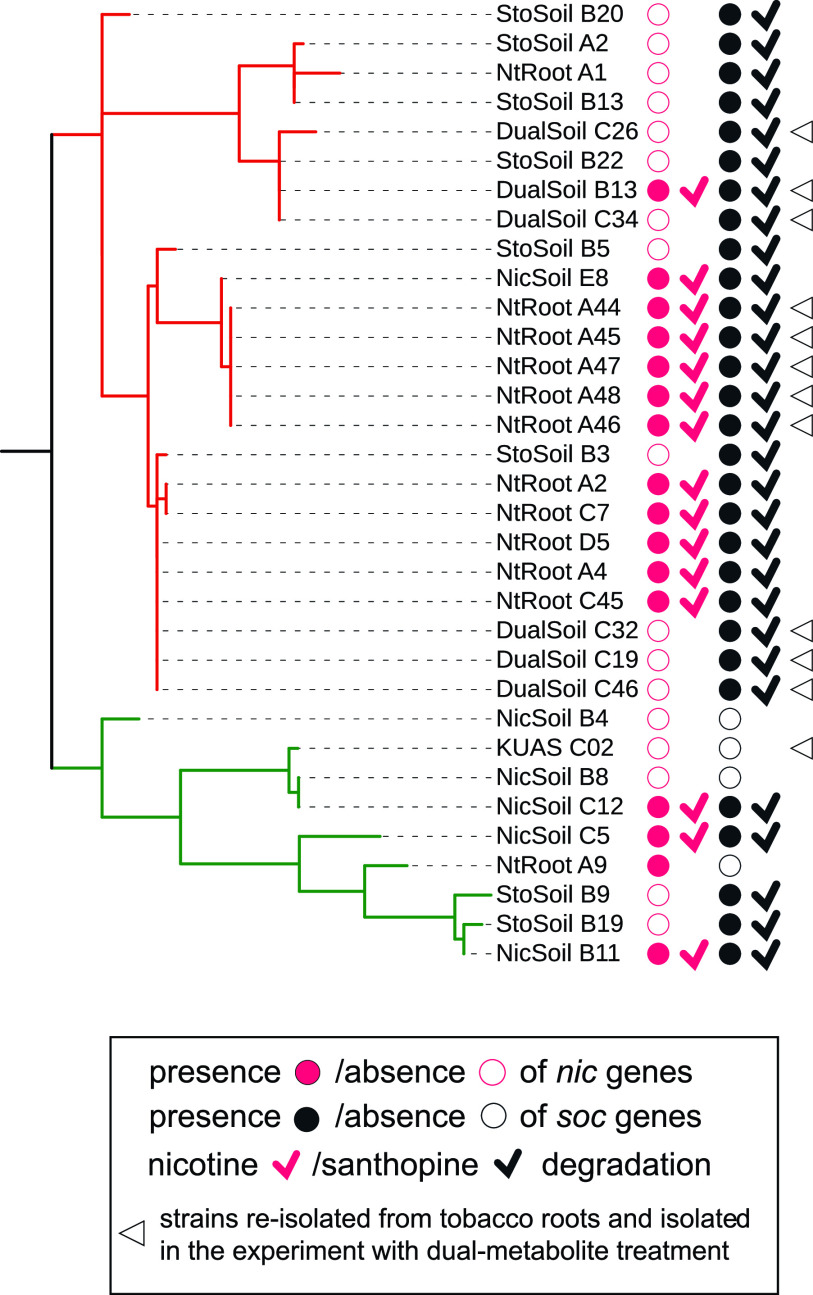
Phylogenetic distribution of catabolic genes and degradation abilities within newly isolated *Arthrobacter* strains. The presence of catabolic genes and degradation ability of santhopine and nicotine are indicated. The branch colors represent the sublineages defined by their AMPHORA phylogeny. The presence and absence of *soc* and *nic* genes are represented by closed and open circles, respectively. The strains reisolated from tobacco roots and isolated in the experiment with dual metabolite treatment are marked with empty arrowheads.

*soc* genes involved in santhopine catabolism were detected in all isolates derived from the tobacco endosphere and santhopine-treated soil, except for NtRootA9, although not necessarily in strains isolated from nicotine-treated soil (Fig. 6; Fig. S3). Bacterial *nic* genes, which are involved in nicotine catabolism, in contrast, were frequently identified in isolates from the tobacco endosphere and nicotine-treated soil but not in isolates from santhopine-treated soil (Fig. 6; Fig. S3). An *in vitro* degradation assay confirmed that the isolates possessing *soc* and/or *nic* genes degraded representative metabolites, except for NtRootA9, which did not degrade nicotine despite the presence of *nic* genes in its genome (Fig. 6). These data revealed that the catabolic potential of these bacterial isolates largely correlated with the presence of representative metabolites in the environment from which they were isolated and suggest a crucial role for the catabolic potential to be competent in the tobacco endosphere. To test this, we carried out a dual-metabolite treatment test of santhopine and nicotine. Similar to monometabolite treatment, dual-metabolite treatment enriched the genus *Arthrobacter* up to 10% of the total bacterial sequence (Fig. S5A and B and Data Set S1D), resulting in a bacterial community closer to that of the tobacco endosphere than to that of the bulk soil (Fig. S5C). We then isolated six and one *Arthrobacter* strains from dual metabolite-treated soils (DualSoil) and mock-treated soil, respectively, and five additional *Arthrobacter* strains from tobacco roots grown in the soil collected from the same agricultural field (Data Set S2D and E). Based on their 16S rRNA gene sequences, all isolates derived from dual-metabolite-treated soils and the tobacco endosphere belonged to sublineage A, and strains newly isolated from tobacco roots formed a monophyletic clade independent from previously isolated strains (Fig. 6). KUAS C02, which was isolated from mock-treated soils, belonged to sublineage B. PCR-based analysis along with an *in vitro* degradation assay identified the metabolic capacity toward santhopine in all isolates derived from the santhopine-containing environment, including the tobacco roots (Fig. 6), whereas the metabolic capacity toward nicotine was detected only in isolates from the tobacco endosphere and one of the isolates from dually treated soils (DualSoil B13) (Fig. 6). Among 33 isolates tested in total, 15 isolates exhibited catabolic capacities toward both metabolites, while 10 out of 12 tested tobacco root-derived isolates were able to catabolize both metabolites, demonstrating that both *soc* and *nic* genes are necessary for niche construction in the tobacco endosphere (*P* = 0.001 by Fisher’s exact test). Importantly, the distributions of *soc* and *nic* genes in sublineages A and B were independent of their origins (Fig. S3), indicating that the individual acquisition of the *nic* or *soc* gene was not associated with niche establishment in the tobacco endosphere but rather was associated with adaptation to the environments with these metabolites. Ancestral character estimation indicated that the most recent common ancestor of sublineage A already possessed the *soc* genes (*P* = 0.983 by MLE), whereas *nic* genes were more recently acquired during the divergence of the sublineages, likely via HGT events (*P *= 0.980 by MLE) (45). These findings illustrate that in addition to the catabolic capacities toward santhopine and nicotine, genes specific to sublineage A are also crucial for bacterial competence in soil and plant environments that are rich in both metabolites. Collectively, our findings imply an evolutionary scenario according to which the presence of the *soc* genes, as well as other ancestral genes specifically found in sublineage A, predisposed ancestral *Arthrobacter* to the colonization of the tobacco endosphere, possibly triggered by HGT of the *nic* genes.

10.1128/mBio.00846-21.6 FIG S5 Bacterial community analysis of dual-metabolite-treated soil. (A) Composition of the 20 most abundant bacterial orders across samples in dual-metabolite-treated soil. (B) Mean relative abundances of the genus *Arthrobacter* in dual-metabolite-treated soil. Each bar represents the mean relative abundance (*n* = 4). Asterisks indicate statistical significance corresponding to the GLM analysis within each data set (α = 0.05). (C) Comparison of bacterial communities in dual-metabolite-treated soils to those of the tobacco endosphere. Weighted UniFrac distances were calculated between dual-metabolite mock treatment and the bulk soil/endosphere of tobacco and between dual-metabolite treatment (500 nmol g^−1^ soil of santhopine and nicotine) and the bulk soil/endosphere of tobacco, respectively. Different letters indicate statistical differences corresponding to a Tukey HSD test (*P < *0.05). Bulk, bulk soil; ES, endosphere. Download FIG S5, PDF file, 0.4 MB. Copyright © 2021 Shimasaki et al. 2021 Shimasaki et al. https://creativecommons.org/licenses/by/4.0/ This content is distributed under the terms of the Creative Commons Attribution 4.0 International license.

## DISCUSSION

We observed a significant association of the host species-specific enrichment of *Arthrobacter* in the tobacco endosphere with the catabolic capacity toward santhopine and nicotine as well as with the subspeciation of sublineage A accompanied by gains and losses of genes yet to be characterized. Although the direct involvement of *soc* and *nic* genes in the tobacco root endophytic competence of *Arthrobacter* remains to be experimentally addressed in a future study, our results revealed an ability of PSMs to modulate the interaction between the host and its root microbiota. Both santhopine and nicotine treatments resulted in an enrichment of the same genus, *Arthrobacter*, in soil bacterial communities, whereas these metabolites differ in biological activities and their corresponding catabolic processes in bacteria. Santhopine is an Amadori compound (46) composed of fructose and glutamine and requires only a few enzymatic steps to be utilized in bacteria (47, 48). The presence of *soc* genes responsible for the catabolism of a wide range of Amadori compounds is limited to a set of bacterial families such as *Rhizobiaceae*, *Microbacteriaceae*, and *Micrococcaceae* (28), rendering santhopine a nutrient source for specific groups of bacteria. It has been reported that Salmonella, a foodborne animal pathogen, has a unique ability to utilize fructose-asparagine, another Amadori compound, as a carbon and nitrogen source to eliminate potential competitors in the inflamed intestine (49). Soc enzymes can metabolize a wide range of Amadori compounds (47, 48), and Amadori compounds occur in various environments, such as foods, decomposing plants, human blood, and plant tissues (50–52). Thus, it is likely that soil-inhabiting *Arthrobacter* species originally acquired *soc* genes to utilize soil-borne Amadori compounds as their nutrients, which in turn provided a competitive advantage against other bacteria in the tobacco endosphere. In contrast, nicotine exhibits antimicrobial activity against various bacteria and fungi (53), and a series of reactions catabolized by the enzymes encoded by the *nic* gene cluster (located on the pAO1 megaplasmid) are required for nicotine catabolism (45). Thus, detoxification and/or utilization of nicotine by Nic enzymes might be beneficial for colonization of the tobacco endosphere, a highly competitive environment with other bacterial species. In addition, *Arthrobacter* AK-YN10 and SJcon strains harboring *nic* genes have been isolated from agricultural soils contaminated by nitrogen-containing pesticides (atrazine and 2-chloro-4-nitrophenol, respectively) (54, 55), pointing to the contribution of *nic* genes to the catabolism of not only nicotine but also other alkaloid-like compounds. Together, it is plausible that the presence of *soc* and *nic* genes in *Arthrobacter* confers competence in environments rich in these classes of metabolites. We demonstrated that almost all strains isolated from tobacco roots possessed *soc* and *nic* genes and exhibited santhopine- and nicotine-catabolic activity *in vitro*, despite their variability within the entire genus, further supporting the importance of these PSMs and the corresponding catabolic capacities in *Arthrobacter* for their specific competence in the tobacco endosphere.

**FIG 7:**
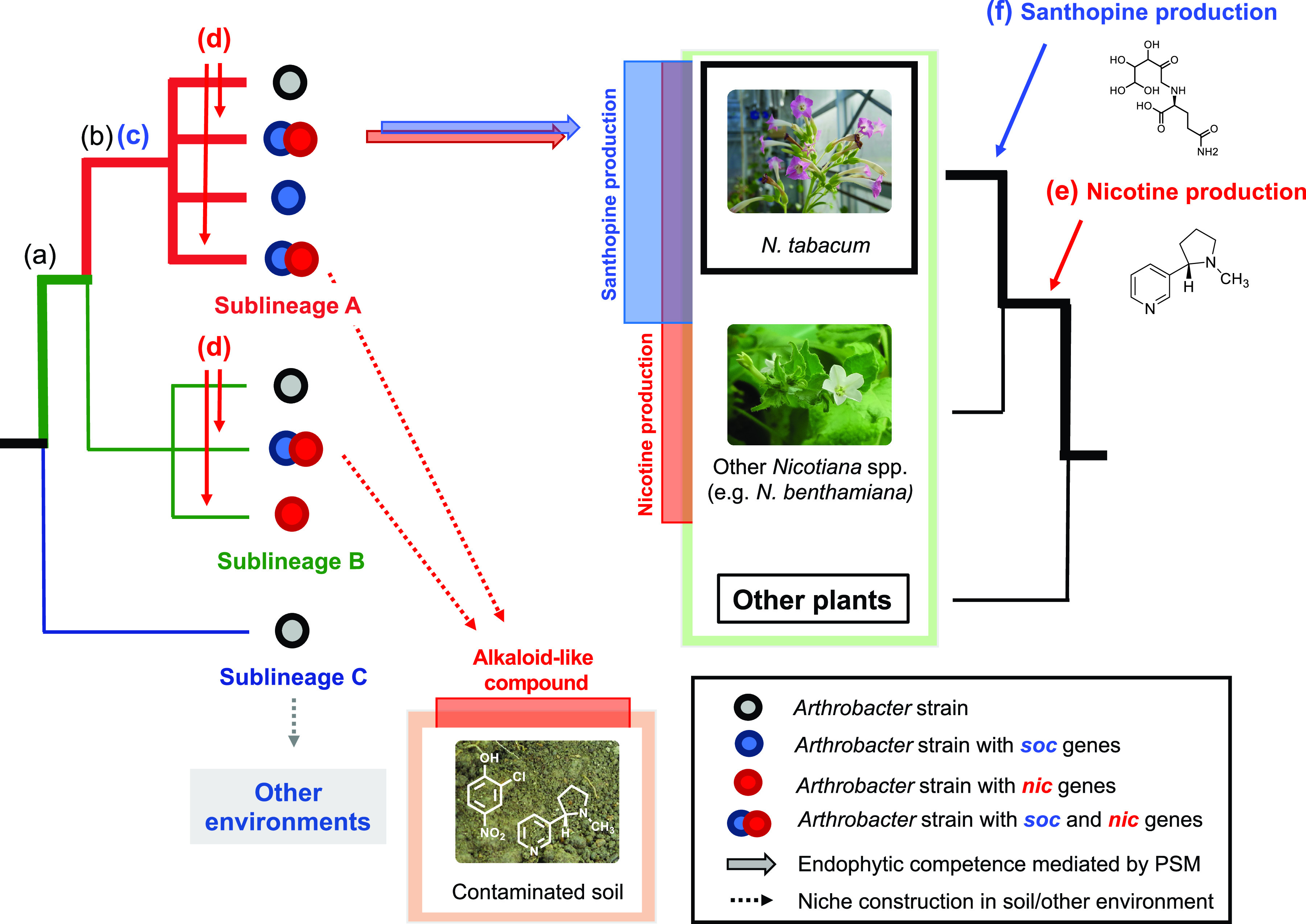
Proposed evolutionary model of a host-specific root microbiota assembly mediated by bacterial catabolism of a combination of PSMs synthesized by the host plant. Anticipated acquisition events of plant and bacterial characteristics related to the interaction between tobacco and *Arthrobacter* are represented by letters. (a) Acquisition of genes required for the plant-associated lifestyle, which are conserved in sublineages A and B. (b) Acquisition of genes specifically enriched in sublineage A. (c) Acquisition of *soc* genes. (d) HGT events of *nic* genes, which have frequently occurred within sublineages A and B. (e) Acquisition of genes required for nicotine production. (f) HGT event of the *mas2* gene for santhopine production. Events b and c may originally be associated with adaptation to soil environments. The cooccurrence of events a to d was observed only in the isolates from tobacco roots. The host-specific enrichment of *Arthrobacter* in tobacco roots was possibly triggered by events d and f in bacteria and plants, respectively.

The importance of santhopine and nicotine for the tobacco-specific endophytic competence of the genus *Arthrobacter* raises the question of how these bacteria might have acquired the ability to catabolize these PSMs and whether this metabolic capacity can alone explain the observed host-specific interactions. Among the three distinctive *Arthrobacter* sublineages defined in this study, sublineages A and B appeared to be better adapted to the soil and/or plant environments, correlated with the presence of genes potentially related to the plant-associated lifestyle. Our data also suggest the presence of uncharacterized functional properties specifically enriched in sublineage A, which are likely to be required for the interaction with tobacco, in addition to the PSM-catabolic activities, but not necessarily for the interaction with plants in general. Notably, isolates that exhibited *in vitro* activity to catabolize both santhopine and nicotine were found in both sublineages A and B, and AK-YN10 and SJcon isolated from soils contaminated by alkaloid-like compounds possessed both *soc* and *nic* genes and belonged to sublineages A and B, respectively (Fig. 6). In contrast, almost all isolates derived from tobacco roots (NtRoot isolates) and the isolate from tobacco waste (M2012083) were specifically found in sublineage A. Combined with the fact that DualSoil isolates do not necessarily possess *nic* genes despite their specific occurrence in sublineage A, it is conceivable that *soc* genes, *nic* genes, and the other genes specifically found in sublineage A are jointly required for the specific enrichment in the tobacco endosphere. Although the functional contribution of the uncharacterized genes specific to sublineage A, as well as of *soc* and *nic* genes, remains to be addressed, we speculate that these genes confer a better efficiency in utilizing carbohydrates and amino acids, including those that can be provided by the degradation of Amadori compounds and alkaloids. The observations that two sets of isolates from tobacco roots from two individual isolation experiments form monophyletic clades that are independent of each other (Fig. 6) and that the isolate from tobacco waste (M2012083) was taxonomically distant from both tobacco-derived clades (Fig. 3) suggest independent acquisition events of the *nic* operon. It appears that the subspeciation of the sublineage as well as the acquisition of *soc* genes in *Arthrobacter* and of nicotine biosynthetic capability in *Nicotiana* plants were earlier than the acquisition of *nic* and *MAS2* genes in *Arthrobacter* and *Nicotiana*, respectively. Overall, our results suggested that the horizontal acquisition of the *nic* genes to catabolize nicotine (45) within *Arthrobacter* sublineage A and the *MAS2* gene to synthesize santhopine in tobacco (20) mediates interactions between this bacterial genus and tobacco plants (Fig. 7). This model implies that the observed interactions between *Arthrobacter* and tobacco may not be a consequence of coevolution between the two partners but rather a result of a process of ecological fitting.

Interestingly, *Arthrobacter* strains isolated from tobacco were able to catabolize both santhopine and nicotine, although these two compounds belong to unrelated classes of PSMs (Fig. 1A). Similarly, both compounds mediate the enrichment of the same bacterial genus in the soil bacterial community. Therefore, the capacity of the host to produce a combination of both compounds contributes to the enrichment of *Arthrobacter* in the root compartment. This finding contrasts with the effect of two PSMs in soybean, isoflavones and soyasaponins, which enriched different bacterial families in soils treated with these metabolites (15, 16). Considering the fact that a plant produces a wide range of lineage-specific PSMs (56), it is plausible that host-specific microbiota assembly is partially achieved by the joint action of multiple PSMs. This mechanism might explain why the disruption of a single metabolic pathway in plants typically results in only minor changes in the root microbiota composition (11–13). Together with previous studies showing the impact of PSMs on the root-associated bacterial community structure (11–13), our findings suggest a mechanistic model of host-specific root microbiota assembly in which the catabolic potential of bacteria toward a cocktail of host-specific PSMs plays a key role.

## MATERIALS AND METHODS

### Chemicals and soil.

Chemicals were obtained from Wako Pure Chemical Industries (Osaka, Japan) or Nacalai Tesque, unless otherwise stated. Field soil (gray lowland soil) was collected from a field at the Kyoto University of Advanced Science (KUAS), Kameoka, Kyoto, Japan (34°99′38″N, 135°55′14″E). Soil chemical and physical properties were described previously (15). Santhopine was synthesized as described previously (20) (see Text S1 in the supplemental material for details).

10.1128/mBio.00846-21.1 TEXT S1 Supplemental methods. Download Text S1, DOCX file, 0.06 MB. Copyright © 2021 Shimasaki et al. 2021 Shimasaki et al. https://creativecommons.org/licenses/by/4.0/ This content is distributed under the terms of the Creative Commons Attribution 4.0 International license.

### Treatment of field soil with plant specialized metabolites.

Three different concentrations of a santhopine or nicotine solution (50, 250, and 1,000 nmol g^−1^ soil) and two different concentrations of a mixed solution of santhopine and nicotine (500 and 1,000 nmol g^−1^ soil each) were prepared. Concentrations of PSMs were set according to our previous report (15). Each metabolite solution was applied to 1 g of air-dried field soil every 3 days five times, as described previously (15). Sterile water was applied as a control. Tubes were incubated at room temperature in the dark. The concentrations of the santhopine or nicotine solution were adjusted every time to keep the soil water content ratio at 30%. After incubation, total DNA was extracted, as described previously (15).

### Sample collection from tobacco roots.

Seeds of N. tabacum cv. Burley 21 (tobacco) were provided by Japan Tobacco, Inc. (Tokyo, Japan). Tobacco seeds were surface sterilized with 70% ethanol (EtOH) for 1 min and 1% sodium hypochlorite (NaClO) for 10 min and rinsed five times with sterile water. Plants were germinated on Murashige and Skoog medium (Wako Pure Chemical Industries) supplemented with 0.8% agar. Plants were grown for 2 weeks in a cultivation room set at 28°C under a light/dark (16/8-h) photoperiod. Tobacco seedlings were then transferred to plastic pots filled with soil collected at the KUAS and grown for 12 weeks in a greenhouse. A 10-week-old plant after transplanting was subjected to reisolation of *Arthrobacter*. The plants were fertilized every week using Hyponex (Hyponex Japan, Osaka, Japan).

The rhizosphere and rhizoplane soils were collected using 250 ml of phosphate-buffered saline (PBS) after removing the loosely attached soil on the roots by gentle shaking, as described previously (6). The roots after sonication were washed with tap water and surface sterilized with 70% EtOH for 1 min and 1% NaClO. The surface-sterilized roots were then washed five times with sterile water and stored at −80°C until DNA extraction. The remaining root tissues were immediately subjected to bacterial isolation.

### Sample collection from field-grown plants.

Tomato seeds (cv. Beni-Suzume) were sown in a culture soil, a 1:1 mixture of vermiculite and Tsuchitaro (Sumitomo Forestry Landscaping, Tokyo, Japan), and grown for 13 days at 28°C under a light/dark (16/8-h) photoperiod. The seedlings were then planted in the field on 9 May 2019. Seeds of soybean (cv. Enrei) and bitter melon were directly sown in the field on 31 May 2019. All plants were sampled on 1 August 2019. After the collection of rhizosphere soils as described above, the roots washed with tap water were subjected to DNA extraction as an endosphere compartment. Soybean and tomato roots for DNA extraction were collected from five and two plants per sample, respectively.

### Bacterial community analysis using 16S rRNA gene amplicon sequencing.

DNA extraction, PCR amplification, and sequence analysis were performed as described previously by Okutani et al. (15). Briefly, the V4 region of the 16S rRNA genes was amplified and sequenced using the MiSeq platform (Illumina). The obtained reads were clustered, classified, and analyzed for α- and β-diversities using the QIIME2 and DADA2 pipelines (57, 58). Detailed methods and computational analyses can be found in Text S1 in the supplemental material.

### Isolation of root- and soil-derived *Arthrobacter* and phylogenic analysis.

Bacterial strains were isolated from the surface-sterilized tobacco roots, and the soils were treated with santhopine or nicotine at 1,000 nmol g^−1^ soil or both metabolites at 1,000 nmol g^−1^ soil. Nonsterilized tobacco roots after sonication, which included rhizoplane and endosphere colonizing bacteria, were used for the reisolation of *Arthrobacter*. The roots were sectioned into smaller fragments and then homogenized with a mortar and pestle in PBS. The santhopine-, nicotine-, and dual-metabolite-treated soils were suspended in distilled water at 0.5 g soil ml^−1^. Homogenates and soil suspensions were diluted and distributed onto isolation media, tryptone yeast extract glucose medium (TYG), yeast extract manitol medium (YEM), M715, and M408, as described previously by Bai et al. (38). Mineral salt buffer (MS) medium (59) with a 1-mg ml^−1^ nicotine solution was also used for isolation from nicotine-treated soil. The media used for the isolation of each bacterial strain are listed in Data Set S2. Plates were incubated for up to 7 days at 28°C. Colonies were picked from plates and subcultured on growth medium (10 g liter^−1^ peptone, 10 g liter^−1^ beef extract, and 5 g liter^−1^ NaCl) and then preserved in a 25% glycerol solution at −80°C. Genomic DNA was extracted by the hot-alkaline DNA extraction method using extraction buffer (25 mM NaOH, 0.2 mM EDTA, 40 mM Tris-HCl [pH 6.8]). The 16S rRNA genes were amplified using primer set 10F (5′-GTTTGATCCTGGCTCA-3′) and 1500R (5′-TACCTTGTTACGACTT-3′). PCR products were purified with the Wizard genomic DNA purification kit (Promega, Madison, WI, USA) according to the manufacturer’s protocol and directly sequenced using primer 10F to identify individual bacterial isolates to the genus level. Fifty-four bacterial isolates assigned to the genus *Arthrobacter* from the bacterial culture collection for further phylogenic analysis (30, 10, 14, and 6 individual *Arthrobacter* strains were isolated from the tobacco endosphere and soils treated with santhopine, nicotine, and dual metabolites, respectively) were then randomly selected and sequenced using primer 1500R. The resulting nearly complete 16S rRNA gene sequences (1,202 bp) were aligned with Clustal Omega 1.2.4 (60), and the resultant alignment was trimmed using trimal 1.4 (61). The 16S rRNA gene sequence data for the reference strains were retrieved from the GenBank database by a BLAST search. Phylogenetic trees were constructed by the MLE method using FastTree version 2.1 (62).

### Comparative genomic analysis of *Arthrobacter*.

Whole-genome sequencing of *Arthrobacter* isolates and their assembly as well as comparative analyses were performed as described previously. Briefly, reads obtained by the Illumina HiSeq 2500 and PacBio Sequell II platforms were assembled and annotated by the A5 and Prokka pipelines, respectively (63, 64). The AMPHORA and OrthoFinder2 pipelines were used to reconstruct phylogenetic relationships and predict orthologous groups, respectively (40, 41). Ancestral character estimation was performed by the ace function of the ape R package using the maximum likelihood estimation approach. A full description of methods and algorithms used in this study can be found in Text S1.

### Santhopine and nicotine degradation assay.

Bacterial strains were cultured until strains reached the stationary phase at 28°C in 2 ml of growth medium as described above. Bacterial cells were collected by centrifugation at 10,000 × *g* for 2 min and washed twice with MS medium. Bacterial pellets were resuspended in 0.5 ml of MS medium with a 5-μg ml^−1^ santhopine solution or a 1-mg ml^−1^ nicotine solution and incubated for 1 to 2 days. After centrifugation at 13,000 × *g* for 5 min, supernatants were collected and stored at −80°C until use. The santhopine content in the supernatants was measured by liquid chromatography-mass spectrometry (LC-MS) (Waters) in the positive electrospray ionization with multiple-reaction monitoring (MRM) mode (see Text S1 for details). The LC mobile phases consisted of water (mobile phase A), acetonitrile (mobile phase B), 2-propanol (mobile phase C), and water containing 4% (vol/vol) formic acid (mobile phase D). The stepwise gradient program was isocratic at 70% mobile phase B, 25% C, and 5% D from 0 to 3 min and isocratic at 47.5% mobile phase A, 35% B, 12.5% C, and 5% D from 3 to 8 min. Nicotine was analyzed by high-performance liquid chromatography (HPLC) as described previously by Häkkinen et al. (65).

### Data availability.

All raw data and scripts as well as intermediate data are available at https://www.mpipz.mpg.de/R_scripts. Additional data related to this paper will be made available upon request.

10.1128/mBio.00846-21.7 FIG S6 Nuclear magnetic resonance (NMR) data for santhopine. Download FIG S6, PDF file, 0.08 MB. Copyright © 2021 Shimasaki et al. 2021 Shimasaki et al. https://creativecommons.org/licenses/by/4.0/ This content is distributed under the terms of the Creative Commons Attribution 4.0 International license.

## References

[1] Hiltner L. 1904. Über neuere Erfahrungen und Probleme auf dem Gebiet der Bodenbakteriologie und unter besonderer Berücksichtigung der Gründüngung und Brache. Arb Dtsch Landwirtschaftlichen Ges 98:59–78.

[2] Miller SB, Heuberger AL, Broeckling CD, Jahn CE. 2019. Non-targeted metabolomics reveals sorghum rhizosphere-associated exudates are influenced by the belowground interaction of substrate and sorghum genotype. Int J Mol Sci 20:431. doi:10.3390/ijms20020431. PMC6358735 30669498

[3] Pétriacq P, Williams A, Cotton A, McFarlane AE, Rolfe SA, Ton J. 2017. Metabolite profiling of non-sterile rhizosphere soil. Plant J 92:147–162. doi:10.1111/tpj.13639. 28742258 PMC5639361

[4] Bulgarelli D, Schlaeppi K, Spaepen S, Ver Loren van Themaat E, Schulze-Lefert P. 2013. Structure and functions of the bacterial microbiota of plants. Annu Rev Plant Biol 64:807–838. doi:10.1146/annurev-arplant-050312-120106. 23373698

[5] Lundberg DS, Lebeis SL, Paredes SH, Yourstone S, Gehring J, Malfatti S, Tremblay J, Engelbrektson A, Kunin V, Del Rio TG, Edgar RC, Eickhorst T, Ley RE, Hugenholtz P, Tringe SG, Dangl JL. 2012. Defining the core *Arabidopsis thaliana* root microbiome. Nature 488:86–90. doi:10.1038/nature11237. 22859206 PMC4074413

[6] Bulgarelli D, Rott M, Schlaeppi K, Ver Loren van Themaat E, Ahmadinejad N, Assenza F, Rauf P, Huettel B, Reinhardt R, Schmelzer E, Peplies J, Gloeckner FO, Amann R, Eickhorst T, Schulze-Lefert P. 2012. Revealing structure and assembly cues for *Arabidopsis* root-inhabiting bacterial microbiota. Nature 488:91–95. doi:10.1038/nature11336. 22859207

[7] Hacquard S, Garrido-Oter R, González A, Spaepen S, Ackermann G, Lebeis S, McHardy AC, Dangl JL, Knight R, Ley R, Schulze-Lefert P. 2015. Microbiota and host nutrition across plant and animal kingdoms. Cell Host Microbe 17:603–616. doi:10.1016/j.chom.2015.04.009. 25974302

[8] Fitzpatrick CR, Copeland J, Wang PW, Guttman DS, Kotanen PM, Johnson MTJ. 2018. Assembly and ecological function of the root microbiome across angiosperm plant species. Proc Natl Acad Sci U S A 115:E1157–E1165. doi:10.1073/pnas.1717617115. 29358405 PMC5819437

[9] Jacoby RP, Chen L, Schwier M, Koprivova A, Kopriva S. 2020. Recent advances in the role of plant metabolites in shaping the root microbiome. F1000Res 9:151. doi:10.12688/f1000research.21796.1. PMC7047909 32148778

[10] Pascale A, Proietti S, Pantelides IS, Stringlis IA. 2019. Modulation of the root microbiome by plant molecules: the basis for targeted disease suppression and plant growth promotion. Front Plant Sci 10:1741. doi:10.3389/fpls.2019.01741. 32038698 PMC6992662

[11] Huang AC, Jiang T, Liu YX, Bai YC, Reed J, Qu B, Goossens A, Nützmann HW, Bai Y, Osbourn A. 2019. A specialized metabolic network selectively modulates *Arabidopsis* root microbiota. Science 364:eaau6389. doi:10.1126/science.aau6389. 31073042

[12] Voges M, Bai Y, Schulze-Lefert P, Sattely ES. 2019. Plant-derived coumarins shape the composition of an *Arabidopsis* synthetic root microbiome. Proc Natl Acad Sci U S A 116:12558–12565. doi:10.1073/pnas.1820691116. 31152139 PMC6589675

[13] Stringlis IA, Yu K, Feussner K, de Jonge R, Van Bentum S, Van Verk MC, Berendsen RL, Bakker P, Feussner I, Pieterse CMJ. 2018. MYB72-dependent coumarin exudation shapes root microbiome assembly to promote plant health. Proc Natl Acad Sci U S A 115:E5213–E5222. doi:10.1073/pnas.1722335115. 29686086 PMC5984513

[14] Harbort CJ, Hashimoto M, Inoue H, Niu Y, Guan R, Rombolà AD, Kopriva S, Voges MJEEE, Sattely ES, Garrido-Oter R, Schulze-Lefert P. 2020. Root-secreted coumarins and the microbiota interact to improve iron nutrition in *Arabidopsis*. Cell Host Microbe 28:825–837.e6. doi:10.1016/j.chom.2020.09.006. 33027611 PMC7738756

[15] Okutani F, Hamamoto S, Aoki Y, Nakayasu M, Nihei N, Nishimura T, Yazaki K, Sugiyama A. 2020. Rhizosphere modelling reveals spatiotemporal distribution of daidzein shaping soybean rhizosphere bacterial community. Plant Cell Environ 43:1036–1046. doi:10.1111/pce.13708. 31875335

[16] Fujimatsu T, Endo K, Yazaki K, Sugiyama A. 2020. Secretion dynamics of soyasaponins in soybean roots and effects to modify the bacterial composition. Plant Direct 4:e00259. doi:10.1002/pld3.259. 32995699 PMC7503093

[17] Levy A, Salas Gonzalez I, Mittelviefhaus M, Clingenpeel S, Herrera Paredes S, Miao J, Wang K, Devescovi G, Stillman K, Monteiro F, Rangel Alvarez B, Lundberg DS, Lu TY, Lebeis S, Jin Z, McDonald M, Klein AP, Feltcher ME, Rio TG, Grant SR, Doty SL, Ley RE, Zhao B, Venturi V, Pelletier DA, Vorholt JA, Tringe SG, Woyke T, Dangl JL. 2017. Genomic features of bacterial adaptation to plants. Nat Genet 50:138–150. doi:10.1038/s41588-017-0012-9. 29255260 PMC5957079

[18] Rodgman A, Perfetti TA. 2009. The chemical components of tobacco and tobacco smoke. CRC Press, Boca Raton, FL. 10.1201/9781420078848.

[19] Jassbi AR, Zare S, Asadollahi M, Schuman MC. 2017. Ecological roles and biological activities of specialized metabolites from the genus *Nicotiana*. Chem Rev 117:12227–12280. doi:10.1021/acs.chemrev.7b00001. 28960061

[20] Chen K, de Borne FD, Julio E, Obszynski J, Pale P, Otten L. 2016. Root-specific expression of opine genes and opine accumulation in some cultivars of the naturally occurring genetically modified organism *Nicotiana tabacum*. Plant J 87:258–269. doi:10.1111/tpj.13196. 27125327

[21] Saito K, Noma M, Kawashima N. 1985. The alkaloid contents of sixty *Nicotiana* species. Phytochemistry 24:477–480. doi:10.1016/S0031-9422(00)80751-7.

[22] Moore LW, Chilton WS, Canfield ML. 1997. Diversity of opines and opine-catabolizing bacteria isolated from naturally occurring crown gall tumors. Appl Environ Microbiol 63:201–207. doi:10.1128/AEM.63.1.201-207.1997. 16535484 PMC1389099

[23] Chilton WS, Stomp AN, Beringue W, Bouzar H, Vaudequin-Dransart V, Petit A, Dessaux Y. 1995. The chrysopine family of Amadori-type crown gall opines. Phytochemistry 40:619–628. doi:10.1016/0031-9422(93)00283-L.

[24] White FF, Garfinkel DJ, Huffman GA, Gordon MP, Nester EW. 1983. Sequences homologous to *Agrobacterium rhizogenes* T-DNA in the genomes of uninfected plants. Nature 301:348–350. doi:10.1038/301348a0.

[25] Suzuki K, Yamashita I, Tanaka N. 2002. Tobacco plants were transformed by *Agrobacterium rhizogenes* infection during their evolution. Plant J 32:775–787. doi:10.1046/j.1365-313x.2002.01468.x. 12472692

[26] Quispe-Huamanquispe DG, Gheysen G, Kreuze JF. 2017. Horizontal gene transfer contributes to plant evolution: the case of *Agrobacterium* T-DNAs. Front Plant Sci 8:2015. doi:10.3389/fpls.2017.02015. 29225610 PMC5705623

[27] Chen K, Dorlhac de Borne F, Szegedi E, Otten L. 2014. Deep sequencing of the ancestral tobacco species *Nicotiana tomentosiformis* reveals multiple T-DNA inserts and a complex evolutionary history of natural transformation in the genus *Nicotiana*. Plant J 80:669–682. doi:10.1111/tpj.12661. 25219519

[28] Marty L, Vigouroux A, Aumont-Nicaise M, Dessaux Y, Faure D, Moréra S. 2016. Structural basis for high specificity of Amadori compound and mannopine opine binding in bacterial pathogens. J Biol Chem 291:22638–22649. doi:10.1074/jbc.M116.745562. 27609514 PMC5077200

[29] Hayashi S, Watanabe M, Kobayashi M, Tohge T, Hashimoto T, Shoji T. 2020. Genetic manipulation of transcriptional regulators alters nicotine biosynthesis in tobacco. Plant Cell Physiol 61:1041–1053. doi:10.1093/pcp/pcaa036. 32191315

[30] Kessler A, Halitschke R, Baldwin IT. 2004. Silencing the jasmonate cascade: induced plant defenses and insect populations. Science 305:665–668. doi:10.1126/science.1096931. 15232071

[31] Baldwin IT, Schmelz EA, Ohnmeiss TE. 1994. Wound-induced changes in root and shoot jasmonic acid pools correlate with induced nicotine synthesis in *Nicotiana sylvestris* spegazzini and comes. J Chem Ecol 20:2139–2157. doi:10.1007/BF02066250. 24242736

[32] Steppuhn A, Gase K, Krock B, Halitschke R, Baldwin IT. 2004. Nicotine’s defensive function in nature. PLoS Biol 2:E217. doi:10.1371/journal.pbio.0020217. 15314646 PMC509292

[33] Ferri S, Kim S, Tsugawa W, Sode K. 2009. Review of fructosyl amino acid oxidase engineering research: a glimpse into the future of hemoglobin A1c biosensing. J Diabetes Sci Technol 3:585–592. doi:10.1177/193229680900300324. 20144298 PMC2769878

[34] Mu Y, Chen Q, Parales RE, Lu Z, Hong Q, He J, Qiu J, Jiang J. 2020. Bacterial catabolism of nicotine: catabolic strains, pathways and modules. Environ Res 183:109258. doi:10.1016/j.envres.2020.109258. 32311908

[35] Busse HJ. 2016. Review of the taxonomy of the genus *Arthrobacter*, emendation of the genus *Arthrobacter sensu lato*, proposal to reclassify selected species of the genus *Arthrobacter* in the novel genera *Glutamicibacter* gen. nov., *Paeniglutamicibacter* gen. nov., *Pseudoglutamicibacter* gen. nov., *Paenarthrobacter* gen. nov. and *Pseudarthrobacter* gen. nov., and emended description of *Arthrobacter roseus*. Int J Syst Evol Microbiol 66:9–37. doi:10.1099/ijsem.0.000702. 26486726

[36] Busse H-J, Wieser M, Buczolits S. 2012. Genus III. *Arthrobacter*, p 578–624. *In* Whitman WB, Goodfellow M, Kämpfer P, Busse H-J, Trujillo ME, Ludwig W, Suzuki K-I, Parte A (ed), Bergey’s manual of systematic bacteriology, vol 5, part A. Springer, New York, NY.

[37] Wippel K, Tao K, Niu Y, Zgadzaj R, Guan R, Dahms E, Zhang P, Jensen DB, Logemann E, Radutoiu S, Schulze-Lefert P, Garrido-Oter R. 2021. Host preference and invasiveness of commensals in the *Lotus* and *Arabidopsis* root microbiota. bioRxiv 10.1101/2021.01.12.426357. PMC8387241 34312531

[38] Bai Y, Müller DB, Srinivas G, Garrido-Oter R, Potthoff E, Rott M, Dombrowski N, Münch PC, Spaepen S, Remus-Emsermann M, Hüttel B, McHardy AC, Vorholt JA, Schulze-Lefert P. 2015. Functional overlap of the *Arabidopsis* leaf and root microbiota. Nature 528:364–369. doi:10.1038/nature16192. 26633631

[39] Durán P, Flores-Uribe J, Wippel K, Zhang P, Guan R, Garrido-Oter R. 2021. Characterization of the *Chlamydomonas reinhardtii* phycosphere reveals conserved features of the plant microbiota. bioRxiv 10.1101/2021.03.04.433956.

[40] Wu M, Eisen JA. 2008. A simple, fast, and accurate method of phylogenomic inference. Genome Biol 9:R151. doi:10.1186/gb-2008-9-10-r151. 18851752 PMC2760878

[41] Emms DM, Kelly S. 2019. OrthoFinder: phylogenetic orthology inference for comparative genomics. Genome Biol 20:238. doi:10.1186/s13059-019-1832-y. 31727128 PMC6857279

[42] Salas ME, Lozano MJ, López JL, Draghi WO, Serrania J, Torres Tejerizo GA, Albicoro FJ, Nilsson JF, Pistorio M, Del Papa MF, Parisi G, Becker A, Lagares A. 2017. Specificity traits consistent with legume-rhizobia coevolution displayed by *Ensifer meliloti* rhizosphere colonization. Environ Microbiol 19:3423–3438. doi:10.1111/1462-2920.13820. 28618121

[43] Wheatley RM, Ford BL, Li L, Aroney STN, Knights HE, Ledermann R, East AK, Ramachandran VK, Poole PS. 2020. Lifestyle adaptations of *Rhizobium* from rhizosphere to symbiosis. Proc Natl Acad Sci U S A 117:23823–23834. doi:10.1073/pnas.2009094117. 32900931 PMC7519234

[44] Sessitsch A, Hardoim P, Döring J, Weilharter A, Krause A, Woyke T, Mitter B, Hauberg-Lotte L, Friedrich F, Rahalkar M, Hurek T, Sarkar A, Bodrossy L, van Overbeek L, Brar D, van Elsas JD, Reinhold-Hurek B. 2012. Functional characteristics of an endophyte community colonizing rice roots as revealed by metagenomic analysis. Mol Plant Microbe Interact 25:28–36. doi:10.1094/MPMI-08-11-0204. 21970692

[45] Igloi GL, Brandsch R. 2003. Sequence of the 165-kilobase catabolic plasmid pAO1 from *Arthrobacter nicotinovorans* and identification of a pAO1-dependent nicotine uptake system. J Bacteriol 185:1976–1986. doi:10.1128/jb.185.6.1976-1986.2003. 12618462 PMC150138

[46] Maillard LC. 1912. Action des acides aminés sur les sucres: formation des mélanoïdines par voie méthodique. C R Acad Sci 154:66–68.

[47] Ferri S, Sakaguchi A, Goto H, Tsugawa W, Sode K. 2005. Isolation and characterization of a fructosyl-amine oxidase from an *Arthrobacter* sp. Biotechnol Lett 27:27–32. doi:10.1007/s10529-004-6312-z. 15685416

[48] Hirokawa K, Kajiyama N. 2002. Recombinant Agrobacterium AgaE-like protein with fructosyl amino acid oxidase activity. Biosci Biotechnol Biochem 66:2323–2329. doi:10.1271/bbb.66.2323. 12506967

[49] Ali MM, Newsom DL, González JF, Sabag-Daigle A, Stahl C, Steidley B, Dubena J, Dyszel JL, Smith JN, Dieye Y, Arsenescu R, Boyaka PN, Krakowka S, Romeo T, Behrman EJ, White P, Ahmer BM. 2014. Fructose-asparagine is a primary nutrient during growth of *Salmonella* in the inflamed intestine. PLoS Pathog 10:e1004209. doi:10.1371/journal.ppat.1004209. 24967579 PMC4072780

[50] Bunn H, Gabbay K, Gallop P. 1978. The glycosylation of hemoglobin: relevance to diabetes mellitus. Science 200:21–27. doi:10.1126/science.635569. 635569

[51] Bechtold U, Rabbani N, Mullineaux PM, Thornalley PJ. 2009. Quantitative measurement of specific biomarkers for protein oxidation, nitration and glycation in *Arabidopsis* leaves. Plant J 59:661–671. doi:10.1111/j.1365-313X.2009.03898.x. 19392687

[52] Troise AD, Wiltafsky M, Fogliano V, Vitaglione P. 2018. The quantification of free Amadori compounds and amino acids allows to model the bound Maillard reaction products formation in soybean products. Food Chem 247:29–38. doi:10.1016/j.foodchem.2017.12.019. 29277225

[53] Pavia CS, Pierre A, Nowakowski J. 2000. Antimicrobial activity of nicotine against a spectrum of bacterial and fungal pathogens. J Med Microbiol 49:675–676. doi:10.1099/0022-1317-49-7-675. 10882095

[54] Sagarkar S, Bhardwaj P, Storck V, Devers-Lamrani M, Martin-Laurent F, Kapley A. 2016. *s*-Triazine degrading bacterial isolate *Arthrobacter* sp. AK-YN10, a candidate for bioaugmentation of atrazine contaminated soil. Appl Microbiol Biotechnol 100:903–913. doi:10.1007/s00253-015-6975-5. 26403923

[55] Arora PK, Jain RK. 2013. *Arthrobacter nitrophenolicus* sp. nov. a new 2-chloro-4-nitrophenol degrading bacterium isolated from contaminated soil. 3 Biotech 3:29–32. doi:10.1007/s13205-012-0066-4. PMC3563742 28324343

[56] Strehmel N, Böttcher C, Schmidt S, Scheel D. 2014. Profiling of secondary metabolites in root exudates of *Arabidopsis thaliana*. Phytochemistry 108:35–46. doi:10.1016/j.phytochem.2014.10.003. 25457500

[57] Bokulich NA, Kaehler BD, Rideout JR, Dillon M, Bolyen E, Knight R, Huttley GA, Gregory Caporaso J. 2018. Optimizing taxonomic classification of marker-gene amplicon sequences with QIIME 2’s q2-feature-classifier plugin. Microbiome 6:90. doi:10.1186/s40168-018-0470-z. 29773078 PMC5956843

[58] Callahan BJ, McMurdie PJ, Rosen MJ, Han AW, Johnson AJ, Holmes SP. 2016. DADA2: high-resolution sample inference from Illumina amplicon data. Nat Methods 13:581–583. doi:10.1038/nmeth.3869. 27214047 PMC4927377

[59] Devers M, Soulas G, Martin-Laurent F. 2004. Real-time reverse transcription PCR analysis of expression of atrazine catabolism genes in two bacterial strains isolated from soil. J Microbiol Methods 56:3–15. doi:10.1016/j.mimet.2003.08.015. 14706746

[60] Sievers F, Wilm A, Dineen D, Gibson TJ, Karplus K, Li W, Lopez R, McWilliam H, Remmert M, Söding J, Thompson JD, Higgins DG. 2011. Fast, scalable generation of high-quality protein multiple sequence alignments using Clustal Omega. Mol Syst Biol 7:539. doi:10.1038/msb.2011.75. 21988835 PMC3261699

[61] Capella-Gutiérrez S, Silla-Martínez JM, Gabaldón T. 2009. trimAl: a tool for automated alignment trimming in large-scale phylogenetic analyses. Bioinformatics 25:1972–1973. doi:10.1093/bioinformatics/btp348. 19505945 PMC2712344

[62] Price MN, Dehal PS, Arkin AP. 2010. FastTree 2—approximately maximum-likelihood trees for large alignments. PLoS One 5:e9490. doi:10.1371/journal.pone.0009490. 20224823 PMC2835736

[63] Tritt A, Eisen JA, Facciotti MT, Darling AE. 2012. An integrated pipeline for de novo assembly of microbial genomes. PLoS One 7:e42304. doi:10.1371/journal.pone.0042304. 23028432 PMC3441570

[64] Seemann T. 2014. Prokka: rapid prokaryotic genome annotation. Bioinformatics 30:2068–2069. doi:10.1093/bioinformatics/btu153. 24642063

[65] Häkkinen ST, Rischer H, Laakso I, Maaheimo H, Seppänen-Laakso T, Oksman-Caldentey K-M. 2004. Anatalline and other methyl jasmonate-inducible nicotine alkaloids from *Nicotiana tabacum* cv. By-2 cell cultures. Planta Med 70:936–941. doi:10.1055/s-2004-832620. 15490322

