# Supplementary figures and images for: Tobacco Root Endophytic Arthrobacter Harbors Genomic Features Enabling the Catabolism of Host-Specific Plant Specialized Metabolites
Source: mBio. 2021 May 28;12(3):e00846-21. doi: 10.1128/mBio.00846-21 (PMC8262997; doi:10.1128/mBio.00846-21)

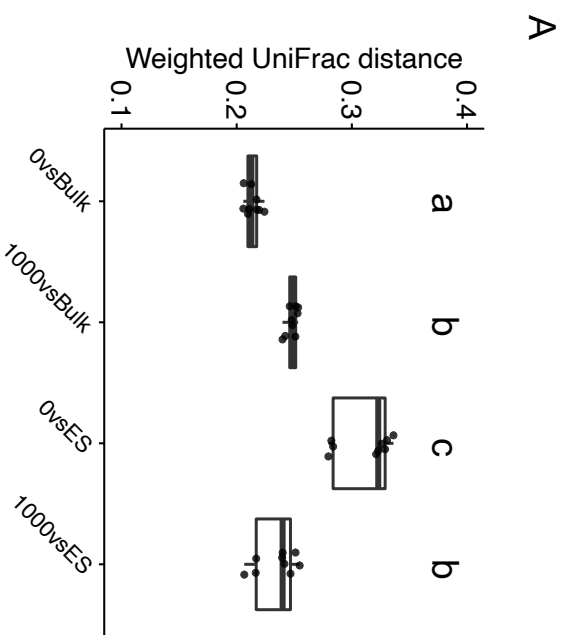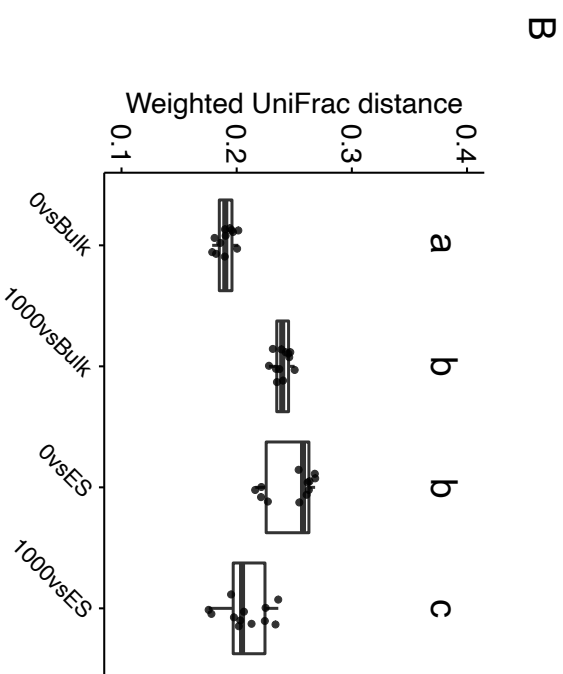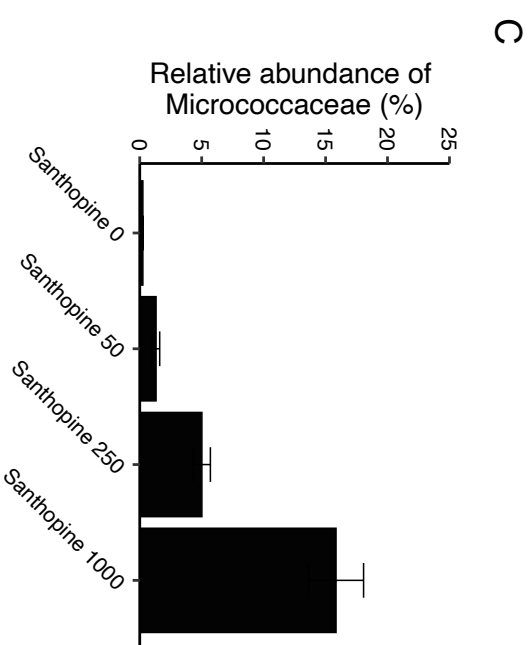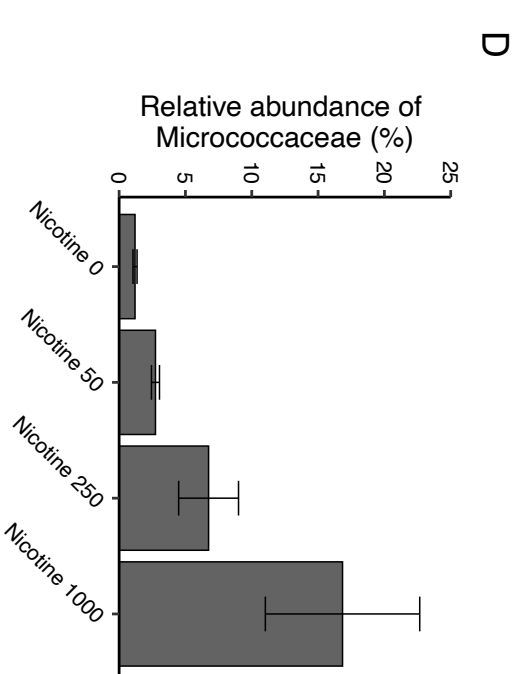

**Fig. S1. Bacterial community analysis of santhopine- and nicotine-treated soil.**

Supplement: FIG S1 [file mbio.00846-21-sf001.pdf]

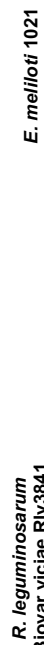

*R. leguminosarum*  
biovar viciae Rlv3841

Supplement: FIG S3 [file mbio.00846-21-sf003.pdf]

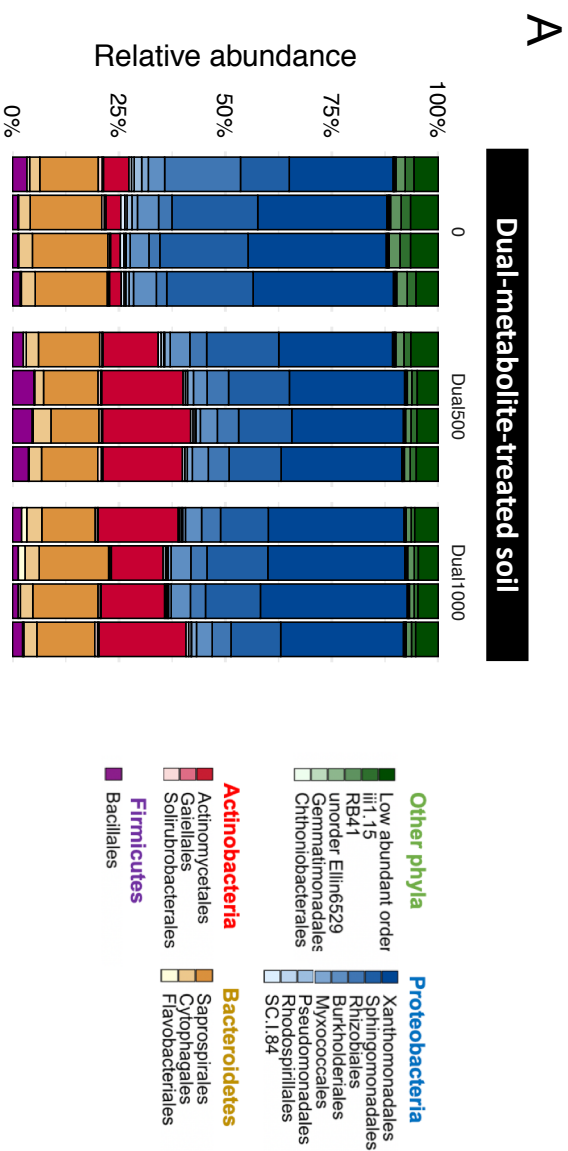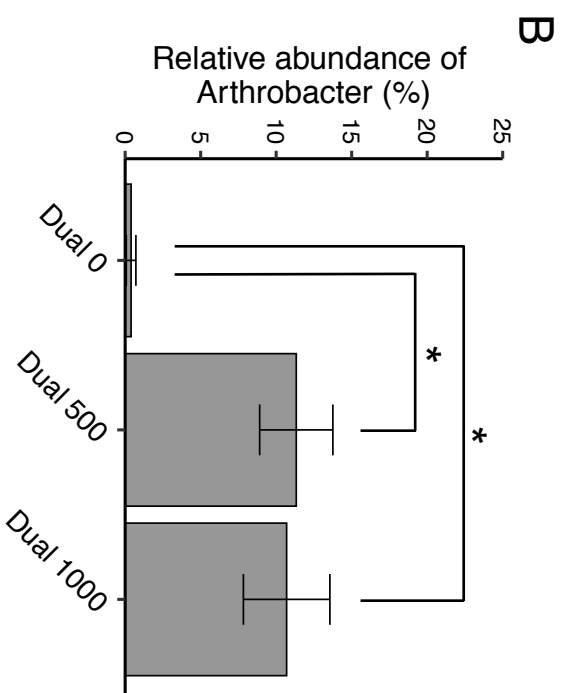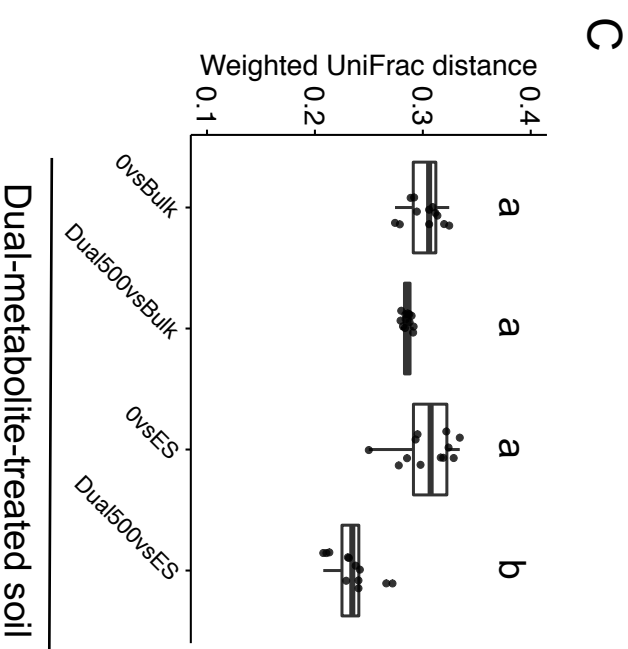

**Fig. S5. Bacterial community analysis of dual-metabolite-treated soil.**

Supplement: FIG S5 [file mbio.00846-21-sf005.pdf]
