# Supplementary material for: Tobacco Root Endophytic Arthrobacter Harbors Genomic Features Enabling the Catabolism of Host-Specific Plant Specialized Metabolites
Source: mBio. 2021 May 28;12(3):e00846-21. doi: 10.1128/mBio.00846-21 (PMC8262997; doi:10.1128/mBio.00846-21)
Supplement: FIG S2 [file mbio.00846-21-sf002.pdf]

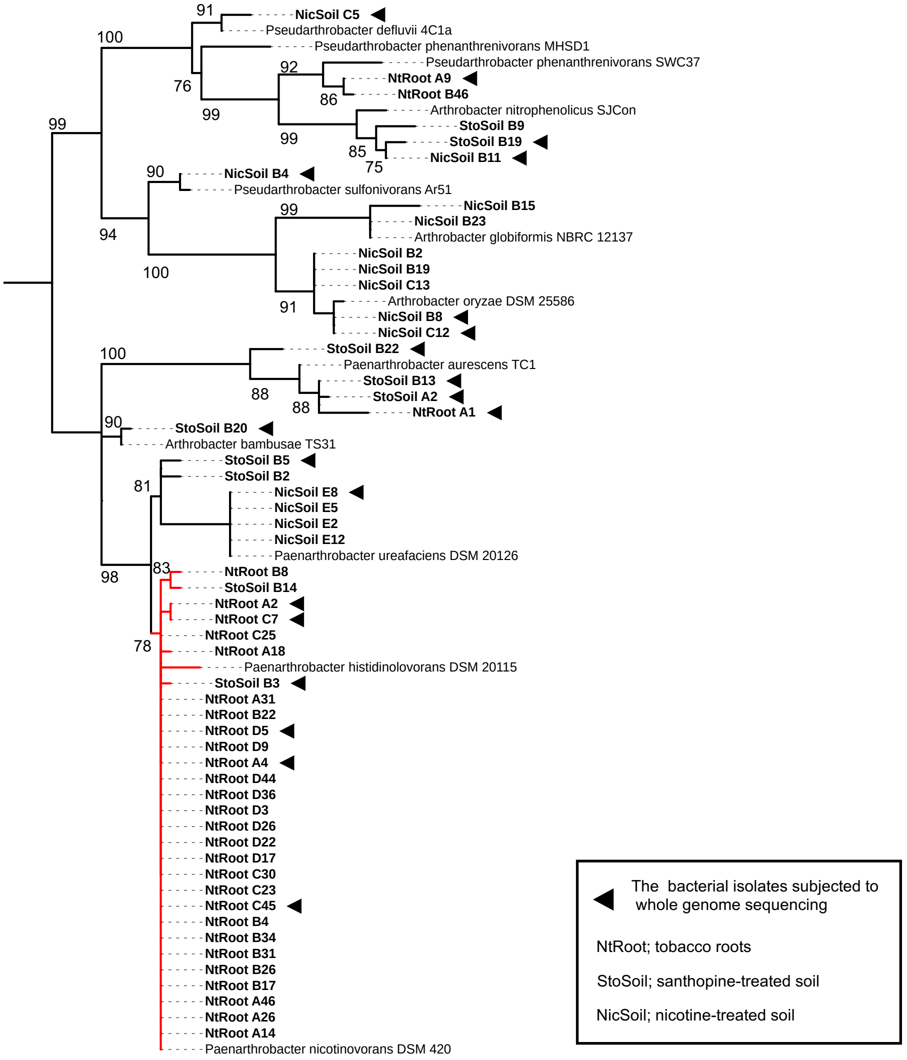

Fig. S2. MLE phylogeny of *Arthrobacter* isolates based on their near-complete *16S* rRNA gene sequences.

Shimasaki *et al.*,
