# Supplementary material for: Tobacco Root Endophytic Arthrobacter Harbors Genomic Features Enabling the Catabolism of Host-Specific Plant Specialized Metabolites
Source: mBio. 2021 May 28;12(3):e00846-21. doi: 10.1128/mBio.00846-21 (PMC8262997; doi:10.1128/mBio.00846-21)
Supplement: TEXT S1 [file mbio.00846-21-s0001.docx]

**Supplemental Methods**

**Chemicals and soil**

Santhopine was synthesized as described previously (1). Glucose (1.35 g) was dissolved in 15 mL AcOH at 95°C, and then L-glutamine (300 mg) was added. The reaction mixture was stirred about 3 min at 95°C. Five times volume of ethyl acetate was added to the reaction mixture and centrifuged at 4000×*g* for 10 min. Then, the precipitate was dried completely with an evaporator and dissolved in 10 mL water. This sample was simply purified by a Sep-Pak C18 20 cc Vac cartridge (Waters, Milford, MA, USA). The flow-through fraction containing santhopine was freeze-dried and dissolved in methanol. Dissolved samples were purified by preparative high-performance liquid chromatography (HPLC) using COSMOSIL Sugar-D packed column (10 mm I.D. × 250 mm; Nacalai Tesque, Kyoto, Japan). Santhopine was eluted at 4.0 mL min^−1^ flow rate with a mobile phase consisted of (A) water and (B) acetonitrile. The stepwise gradient program was as follows: 95% A, 0 to 5 min; isocratic at 50% A, 5 to 15 min. Absorbance at 210 nm was monitored. Fractions containing santhopine were pooled and completely dried by an evaporator and freeze-dried.

The structure of the synthesized sample was confirmed by liquid chromatography-mass spectrometry (LC-MS) analysis and nuclear magnetic resonance (NMR) spectroscopies (^1^H and ^13^C). LC-MS analysis was performed using an ACQUITY UPLC H-Class system (Waters) with an ACQUITY UPLC BEH amide column (1.7 μm, 2.1 × 100 mm; Waters) at 40°C and an SQ Detector 2 (Waters). The injection volume of each sample was 2 μL and the flow rate was 0.2 ml min^−1^. The LC mobile phase consisted of (A) water, (B) acetonitrile, and (C) water containing 4% (v/v) formic acid. The elution program was a linear gradient of 90 to 50% A supplied with 5% C at 0 to 15 min. Santhopine was analysed in a positive electrospray ionization with the following conditions: cone voltage, 25 V; capillary voltage, 3.15 kV; source temperature, 150 °C; desolvation gas temperature, 400°C; and nebulizer and desolvation N_2_ gas flow rates, 50 and 800 L h^−1^, respectively. Santhopine was detected using selected ion recording (SIR) mode with *m/z* 309.2 and multiple reaction monitoring (MRM) mode with the following conditions: transition, *m/z* 309.2 > 130.1; cone voltage, 25 V; and collision energy, 20 eV. Data acquisition and analysis were performed using MassLynx 4.1 software (Waters).

^1^H and ^13^C NMR spectra were measured at 25°C on a 400 MHz and 100 MHz instrument in deuterated water, respectively. ^1^H NMR (400 MHz, D_2_O, 25 °C): δ 3.92–3.89 (m, 2H, H(3′), H(6′)), 3.76 (dd, 1H, H(4′)), 3.66–3.59 (m, 3H, H(3′), H(6′), CH(4)), 3.20–3.18 (m, 2H, CH_2_(1′)), 2.40–2.38 (m, 2H, CH_2_(2)), 2.01–1.99 (m, 2H, CH_2_(3)). ^13^CNMR (100 MHz, D_2_O, 25 °C) : δ 177.8 (C=O(C-1)), 172.8 (C=O(C-5)), 95.3 (C-2′), 69.9 (C-4′), 69.3 (C-5′), 68.9 (C-3′), 62.6 (CH(C-4)), 63.9 (C-6′), 52.6 (CH_2_(C-1′)), 31.1 (CH_2_(C-2)), 24.9 (CH_2_(C-3)) (Fig. S6).

Chemicals were obtained from Wako Pure Chemical Industries (Osaka, Japan) or Nacalai Tesque, unless otherwise stated. Field soil was collected from a field at the Kyoto University of Advanced Science (KUAS), Kameoka, Kyoto, Japan (34°99′38″N, 135°55′14″E), as described previously (2).

**Bacterial community analysis using *16S* rRNA amplicon sequencing**

For the amplicon sequencing of the *16S* rRNA gene, total DNA was extracted from the soil and plant samples using DNeasy PowerSoil Kit (Qiagen) according to the manufacturer’s protocol. Extracted DNA was quantified using Qubit Quantification Platform dsDNA HS Assay Kit (Invitrogen). PCR amplification of the region V4 of *16S* rRNA was performed with KOD Fx Neo Polymerase using first PCR primers 515F

(5′-ACACTCTTTCCCTACACGACGCTCTTCCGATCT-GTGCCAGCMGCCGCGGTAA-3′) and 806R

(5′-GTGACTGGAGTTCAGACGTGTGCTCTTCCGATCT-GGACTACHVGGGTWTCTAAT-3′) consisted of the Illumina (San Diego, CA, USA) paired-end adapter sequences (underlined) and *16S* rRNA gene-specific sequences. The first PCR mixture for the soil DNA amplification consisted of 10 ng DNA template, 12.5 μL of 2× reaction buffer, 5 μL of 10 mM dNTP, 0.75 μL of 10 μM primers (each), 0.25 μL of KOD Fx Neo (Toyobo, Osaka, Japan), and 0.75 μL of nuclease and DNA-free water. The thermal programs of the first PCR for soil samples were as follows: 2 min at 94°C, 20 cycles of 10 s at 98°C, 30 s at 50°C, and 30 s at 68°C. For the first amplification of the plant-extracted DNA, 25 μM peptide nucleic acids, mPNA and pPNA, were applied: mPNA
(N-term-GGCAAGTGTTCTTCGGA-C-term) and pPNA

(N-term-GGCTCAACCCTGGACAG-C-term), respectively (Panagene Inc., Daejeon, South Korea), to block the amplification of contaminating sequences from a eukaryotic host. PCR reaction was conducted using the following thermal programs: 2 min at 94°C, 25 cycles of 10 s at 98°C, 10 s at 78°C, 30 s at 50°C, and 30 s at 68°C. The amplified fragments were purified using Ampure magnetic beads (Beckman Coulter, Danvers, MA, USA) according to the manufacturer’s protocol. The second PCR mixture content was the same as the first PCR mixture for the soil DNA using primers provided from FASMAC Co., Ltd. (Kanagawa, Japan) and amplified using the following thermal conditions: 2 min at 94°C, 9 to 10 cycles of 10 s at 98°C, 30 s at 50°C, and 30 s at 68°C. The purification and quantification of PCR products were performed as described above. The PCR product mixture was sent to FASMAC to acquire 2 × 250 bp paired-end sequences using the MiSeq platform (Illumina).

Sequence data for the amplicons were analysed using the QIIME2 platform, version 2019.04 (3). For all paired reads, the first 20 bases of both sequences were trimmed (to remove primer sequences), and the bases after 220 were truncated (to remove low-quality sequence data). Potential amplicon sequencing errors were corrected using DADA2 to produce an ASV dataset (4). Obtained ASVs were aligned using MAFFT (4), and a phylogenetic tree was contracted using FastTree 2 (5). Each ASV was assigned using a naïve Bayes classifier from the Greengenes release 13_8 dataset (3), and then the reads for chloroplasts or mitochondria were removed. Obtained data was normalized by rarefying to 6,000 reads per samples for community analysis. The calculation of UniFrac distances, PCoA analysis for β-diversity, and Adonis PERMANOVA test for β group significance was performed using the QIIME2 platform. The Wilcoxon’s rank-sum test and TukeyHSD test comparing weighted UniFrac distance was performed using R software. Statistical analyses of differentially abundant families and orders were performed using the edgeR library (6) by fitting a negative binomial generalized linearized model to the ASVs. The sequence dataset supporting the results of this study was submitted to the DNA Data Bank of Japan (<https://www.ddbj.nig.ac.jp>) (PRJDB11062).

**Whole genome sequencing of *Arthrobacter* isolates**

The genomic DNA of *Arthrobacter* isolates was extracted as described by Hahn and Hennecke with a modification (7). Briefly, the cells were lysed by the addition of 20 mg lysozyme, 10% sodium dodecyl sulfate, and proteinase K (20 mg mL^−1^) and incubated for 1 h at 37°C. The cell lysate was forced through a syringe (21G×1-1/2″ RB; Terumo Corporation, Tokyo, Japan) and extracted with phenol and chloroform. The genomic DNA was dissolved in TE buffer and kept at 4°C. For SMRTbell library preparation, each genomic DNA was fragmented at 20 kbp using a Megaruptor2 [Diagenode, Seraing (Ougrée), Belgium], and the library was constructed using SMRTbell Express Template Prep Kit 2.0 according to the manufacturer’s protocol (Pacific Biosciences, Menlo Park, CA, USA). The barcodes were attached to each fragmented genome, and the samples were pooled and cut off at 15 kbp using the BluePippin size selection system (Sage Science, Cummings Center Beverly, MA, USA). The genomic library was sequenced on a single PacBio sequel II system 2.0 cell. Genomes were assembled with HGAP4 via SMRTlink (version 8.0.0) using the specified genome sizes. The sequence dataset was submitted to the DNA Data Bank of Japan (<https://www.ddbj.nig.ac.jp>) (PRJDB11299).

**Whole genome sequencing of *Arthrobacter* isolates from *L. japonicus* and *C. reinhardtii***

Genomic DNA of bacterial strains isolated from *L. japonicus* rhizosphere and *C. reinhardtii* phycosphere samples was extracted and purified as described previously (8) and sequenced using the Illumina HiSeq 2500 at the Max Planck Genome Center (Cologne, Germany) with an insert size of 350 bp and an approximate depth of 5M reads per strain. Sequences were quality filtered by passing short reads through a quality and length-trimming filter using Trimmomatic (9) with the default parameters and subsequently assembling them using the A5 pipeline (10).

**Genome annotation and orthology inference**

Public genome sequences of *Arthrobacter* were retrieved from the Integrated Microbial Genomes & Microbiomes system (<https://img.jgi.doe.gov>) and the National Center for Biotechnology Information (NCBI; <https://www.ncbi.nlm.nih.gov>). Using a total of 99 genomes, putative protein-coding sequences were predicted using Prokka (11). The annotation of candidate ORFs was then conducted using the KO database (12) by building a blast database using a de-replicated set representative sequences for each KO which was subsequently added to the Prokka pipeline as a custom Bacterial database. *De novo* orthology prediction was performed using OrthoFinder2 to obtain OGs (13).

**Comparative genomics and ancestral character reconstruction**

For high-resolution phylogenetic inference of genomes, well-conserved (present in all genomes), single-copy bacterial genes were extracted using AMPHORA (14). The obtained sequences were aligned independently for each gene by Clustal Omega 1.2.4. Aligned sequences were trimmed and concatenated to infer a phylogenetic tree by an MLE method using FastTree version 2.1. PCoA of a functional diversity between sequenced isolates was performed, as described previously (8). Briefly, we generated a presence/absence profile of each KO and OG group was generated for each genome in the dataset. A dissimilarity matrix was generated based on Pearson’s correlation coefficients between each pair of genomes.

To avoid conflicts in the inference of ancestral characters, polytomies in the trees were resolved by inserting branches of zero length. Given the matrix of phyletic patterns based on KOs or OGs and the estimated species tree, an MLE approach (15) was used to estimate the ancestral state using the *ace* function implemented in the ape package (16) for R software. Ancestral characters of each *Arthrobacter* sublineage were defined as genes with likelihood values > 0.8 at a given node. The functions of identified OGs were further inferred by blasting representative amino acid sequences against the proteome dataset of the family Micrococcaceae at the NCBI (setting: *E*-value thread = 1 × 10^−50^).

To identify homologous genes essential for the root colonization of rhizobia, deduced amino acid sequences of these genes were blasted against the proteome predicted by the *Arthrobacter* genome sequences (>50% query sequence coverage; *E*-value thread > 1 × 10^−5^). The presence of a santhopine catabolism operon was assessed by confirming the presence of *socD* from *Arthrobacter aurescens* TC1 (ABM07460.1, blast setting: *E*-value thread > 3 × 10^−100^), as other *soc* components show high similarities to genes of amino acid metabolism genes, which might overestimate its prevalence. The presence of the *nic* gene cluster was surveyed using *nic* genes of *A. nicotinovorans* DSM420 (*ndhL*, CAD47952.1; *ndhM*, CAD47954.1; *6Hlno*, CAA11306.1; *Ponh*, CAD47941.1; *kdhL*, CAD47940.1; *kdhM*, CAD47951.1; *dhph*, CAD47937.1) as query sequences (blast setting: *E*-value thread = 0). *ndhS* (CAD47953.1) and *kdhS* (CAD47946.1) genes were excluded due to their relatively short amino acid sequence lengths (156 and 160 amino acids, respectively). Phylogenetic trees and associated data were visualized using iTOL version 5 (<https://itol.embl.de>).

**PCR detection of *soc* and *nic* genes**

*SocA* and *ndhL* genes were amplified using *socA*_F (5′-CATSGGVGANCCCGGGAACCAYTT-3′) and *socA*_R (5′-ATCGGSATYACSGACAAGCGCAA-3′), and *ndhL*_F (5′-AACCAAACGGAGATAGCGCA-3′) and *ndhL*_R (5′-GGGAATCAGCGTTTTCGTCG-3′) primers sets, repectively. In the case of positive result, PCR products were purified with Wizard Genomic DNA Purification Kit (Promega, Madison, WI, USA) according to the manufacturer’s protocol and directly sequenced using *socA*_F or *ndhL*_F primer.

**References**

1. Chen K, de Borne FD, Julio E, Obszynski J, Pale P, Otten L. 2016. Root-specific expression of opine genes and opine accumulation in some cultivars of the naturally occurring genetically modified organism *Nicotiana tabacum*. Plant J 87:258-69.

2. Okutani F, Hamamoto S, Aoki Y, Nakayasu M, Nihei N, Nishimura T, Yazaki K, Sugiyama A. 2020. Rhizosphere modelling reveals spatiotemporal distribution of daidzein shaping soybean rhizosphere bacterial community. Plant Cell Environ 43:1036-1046.

3. Bokulich NA, Kaehler BD, Rideout JR, Dillon M, Bolyen E, Knight R, Huttley GA, Gregory Caporaso J. 2018. Optimizing taxonomic classification of marker-gene amplicon sequences with QIIME 2's q2-feature-classifier plugin. Microbiome 6:90.

4. Callahan BJ, McMurdie PJ, Rosen MJ, Han AW, Johnson AJ, Holmes SP. 2016. DADA2: High-resolution sample inference from Illumina amplicon data. Nat Methods 13:581-3.

5. Price MN, Dehal PS, Arkin AP. 2010. FastTree 2--approximately maximum-likelihood trees for large alignments. PLoS One 5:e9490.

6. Robinson MD, McCarthy DJ, Smyth GK. 2010. edgeR: a Bioconductor package for differential expression analysis of digital gene expression data. Bioinformatics 26:139-40.

7. Hahn M, Hennecke H. 1984. Localized mutagenesis in *Rhizobium japonicum*. Mol Gene Genet 193:46-52.

8. Bai Y, Müller DB, Srinivas G, Garrido-Oter R, Potthoff E, Rott M, Dombrowski N, Münch PC, Spaepen S, Remus-Emsermann M, Hüttel B, McHardy AC, Vorholt JA, Schulze-Lefert P. 2015. Functional overlap of the *Arabidopsis* leaf and root microbiota. Nature 528:364-9.

9. Bolger AM, Lohse M, Usadel B. 2014. Trimmomatic: a flexible trimmer for Illumina sequence data. Bioinformatics 30:2114-20.

10. Tritt A, Eisen JA, Facciotti MT, Darling AE. 2012. An integrated pipeline for de novo assembly of microbial genomes. PLoS One 7:e42304.

11. Seemann T. 2014. Prokka: rapid prokaryotic genome annotation. Bioinformatics 30:2068-9.

12. Kanehisa M, Sato Y, Kawashima M, Furumichi M, Tanabe M. 2016. KEGG as a reference resource for gene and protein annotation. Nucleic Acids Res 44:D457-62.

13. Emms DM, Kelly S. 2019. OrthoFinder: phylogenetic orthology inference for comparative genomics. Genome Biol 20:238.

14. Wu M, Eisen JA. 2008. A simple, fast, and accurate method of phylogenomic inference. Genome Biol 9:R151.

15. Pagel M. 1994. Detecting correlated evolution on phylogenies: a general method for the comparative analysis of discrete characters. Proc Biol Sci 255:35-45.

16. Paradis E, Claude J, Strimmer K. 2004. APE: Analyses of phylogenetics and evolution in R language. Bioinformatics 20:289-90.

17. Häkkinen ST, Rischer H, Laakso I, Maaheimo H, Seppänen-Laakso T, Oksman-Caldentey KM. 2004. Anatalline and other methyl jasmonate-inducible nicotine alkaloids from *Nicotiana tabacum* cv. By-2 cell cultures. Planta Med 70:936-41.
