# Supplementary material for: Tobacco Root Endophytic Arthrobacter Harbors Genomic Features Enabling the Catabolism of Host-Specific Plant Specialized Metabolites
Source: mBio. 2021 May 28;12(3):e00846-21. doi: 10.1128/mBio.00846-21 (PMC8262997; doi:10.1128/mBio.00846-21)
Supplement: FIG S6 [file mbio.00846-21-sf006.pdf]

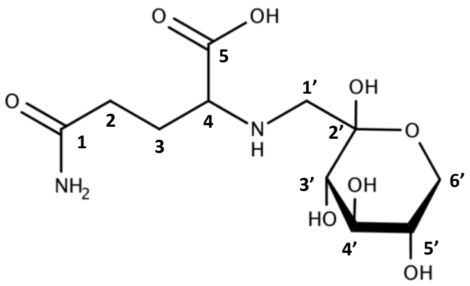

Position	Compound A[a]		Santhopine [a, b]	
	δ_C (ppm)	δ_H multiplicity	δ_C (ppm)	δ_H multiplicity
1	177.8		177.8	
2	31.1	2.40-2.38 m, 2H	31.0	2.50-2.48 m, 2H
3	24.9	2.01-1.99 m, 2H	24.8	2.20-2.13 m, 2H
4	62.6	3.66-3.59 m, 1H (in 3H)	62.6	3.77-3.71 m, 1H (in 3H)
5	172.8		172.7	
1'	52.6	3.20-3.18 m, 2H	52.5	3.32-3.25 m, 2H
2'	95.3		95.3	
3'	68.9	3.92-3.89 m, 1H (in 2H)	68.8	4.02-3.98 m, 1H (in 2H)
4'	69.9	3.76 dd, 1H	69.8	3.77-3.71 m, 1H (in 3H)
5'	69.3	3.66-3.59 m, 1H (in 3H)	69.3	3.85 m, 1H
6'	63.9	3.66-3.59 m, 1H (in 3H)	63.8	3.77-3.71 m, 1H (in 3H)
		3.92-3.89 m, 1H (in 2H)		4.02-3.98 m, 1H (in 2H)

[a] NMR data for compound A or santhopine in D_2O

[b] Chen et al. 2016 *(16)*

Fig. S6. NMR data for santhopine.
